# Evolution of gene structure in the conifer *Picea glauca*: a comparative analysis of the impact of intron size

**DOI:** 10.1186/1471-2229-14-95

**Published:** 2014-04-16

**Authors:** Juliana Stival Sena, Isabelle Giguère, Brian Boyle, Philippe Rigault, Inanc Birol, Andrea Zuccolo, Kermit Ritland, Carol Ritland, Joerg Bohlmann, Steven Jones, Jean Bousquet, John Mackay

**Affiliations:** 1 Center for Forest Research and Institute for Systems and Integrative Biology, 1030 rue de la Médecine, Université Laval, Québec, QC G1V 0A6, Canada; 2 Gydle Inc., Québec, QC, Canada; 3 Michael Smith Laboratories, University of British Columbia, Vancouver, BC V6T 1Z4, Canada; 4 Applied Genomics Institute, Udine 33100, Italy; 5 Institute of Life Sciences, Scuola Superiore Sant’Anna, Pisa 56127, Italy; 6 Department of Forest Sciences, University of British Columbia, Vancouver, BC V6T 1Z4, Canada; 7 Canada Research Chair in Forest Genomics, Université Laval, Québec, QC G1V 0A6, Canada

**Keywords:** Genome size, *Pinus taeda*, BAC, Repeat elements, Gymnosperms, Gene expression

## Abstract

**Background:**

A positive relationship between genome size and intron length is observed across eukaryotes including Angiosperms plants, indicating a co-evolution of genome size and gene structure. Conifers have very large genomes and longer introns on average than most plants, but impacts of their large genome and longer introns on gene structure has not be described.

**Results:**

Gene structure was analyzed for 35 genes of *Picea glauca* obtained from BAC sequencing and genome assembly, including comparisons with *A. thaliana*, *P. trichocarpa* and *Z. mays*. We aimed to develop an understanding of impact of long introns on the structure of individual genes. The number and length of exons was well conserved among the species compared but on average, *P. glauca* introns were longer and genes had four times more intronic sequence than *Arabidopsis,* and 2 times more than poplar and maize. However, pairwise comparisons of individual genes gave variable results and not all contrasts were statistically significant. Genes generally accumulated one or a few longer introns in species with larger genomes but the position of long introns was variable between plant lineages. In *P. glauca,* highly expressed genes generally had more intronic sequence than tissue preferential genes. Comparisons with the *Pinus taeda* BACs and genome scaffolds showed a high conservation for position of long introns and for sequence of short introns. A survey of 1836 *P. glauca* genes obtained by sequence capture mostly containing introns <1 Kbp showed that repeated sequences were 10× more abundant in introns than in exons.

**Conclusion:**

Conifers have large amounts of intronic sequence per gene for seed plants due to the presence of few long introns and repetitive element sequences are ubiquitous in their introns. Results indicate a complex landscape of intron sizes and distribution across taxa and between genes with different expression profiles.

## Background

Many factors related to genome size, recombination rate, expression level, and effective population size, among others, have been proposed to affect the evolution of gene structure [1-4]. At the molecular level, genome size variations may result from mobile or transposable elements (TEs), whole genome duplication events, and polyploidization events, among others. Comparative studies have shown that intron lengths and the abundance of mobile elements directly correlate with genome size, such that large genomes have longer introns and a higher proportion of mobile elements [1]. Mobile elements also impact gene structure and function as they can insert into genes, including introns and exons, and thus contribute to the evolution of genes.

Conifer trees have very large genomes ranging from 18 to 35 Gbp [5] that are composed of a large fraction of repetitive sequences [6,7]. New insight into plant genome evolution are expected from the unique structure and history of conifer genomes [8], which may contribute to a broader understanding of the relationships between gene structure and genome architecture. Draft genome assemblies were recently reported for the European *Picea abies* (Norway spruce) [9] as well as the North American species *Picea glauca* (white spruce) [10] and *Pinus taeda* (loblolly pine) [11,12]. Nystedt et al. [9] reported that Norway spruce and other conifers accumulate long introns and showed that some introns can be very long (>10 Kbp) compared to other plant species.

A positive relationship between genome size and intron length has been observed in broad phylogenetic studies [2,13,14] including between recently diverged *Drosophila* species harboring considerable difference in genome size, where *D. viliris* had longer introns than *D. melanogaster*[15]. In plants, a few studies investigated this question within angiosperms, indicating that genome size is not necessarily a good predictor of intron length [16,17] although a general trend is observed. For instance, *Arabidopsis thaliana, Populus trichocarpa*, *Zea mays* have well characterized genomes that range in size from 125 Mbp to 2.3 Gbp; their average exons sizes are between 250 and 259, whereas their introns sizes are 168 bp, 380 bp and 607 bp on average, respectively [18-20]. The length of introns may depend upon gene function and expression level; however, there is considerable debate surrounding this issue when it comes to plant genomes. In *Oryza sativa* and *A. thaliana* it was found that highly expressed genes contained more and longer introns than genes expressed at a low level [21], which is in contrast to findings in *Caenorhabditis elegans* and *Homo sapiens*[4].

Transposable elements are among the factors that may influence the evolution of intron size, as they represent the major component of plant genomes [22]. In *Vitis vinifera*, transposable elements comprise 80% of long introns [17]. In many plants, LTR-RT represent a large fraction of the genome but are more abundant in gene poor regions of the genome; therefore, their impact on the evolution of gene structure may actually be lesser than other classes of transposable elements such as MITEs [23] and helitrons, both of which are known to insert into or close to genes [24].

To date, studies related to genome size and the evolution of plant introns have primarily involved angiosperms (flowering plants), many of which have genomes under 1 Gbp. More recently, the *Picea abies* and *Pinus taeda* genomes were shown to have among the largest average introns size [9,12]. We aimed to develop an understanding of the gene structure in conifers through a detailed analysis of individual genes with a particular emphasis on the potential impact of long introns on gene structure trough comparative analyses. An underlying question relates to potential impacts on gene expression; therefore, our analyses took into account their expression profiles. Gene structure was analyzed in two conifers (*P. glauca* and *P. taeda*) and three angiosperms. We explored three main hypothesis: (1) Intron length is the major type of variation affecting gene structure in conifers compared to other plant species; (2) there is a positive relationship between genome size and intron length in *P. glauca* compared to *A. thaliana*, *Z. mays* and *P. trichocarpa*; (3) *P. glauca* and *P. taeda* present a conserved gene structure despite the fact that they diverged over 100 MYA in keeping with their low rate of genome evolution [8].

We present a detailed analysis of gene structure for 35 genes from the conifer *Picea glauca* obtained from BAC sequencing and genome assembly and comparative analyses with *A. thaliana*, *P. trichocarpa* and *Z. mays*. Our study also included the analysis of nearly 6000 gene sequences obtained from sequence capture aiming to explore the potential impact of repetitive sequences on intron size in *P. glauca*. Our findings show that intron size and the position of long introns within genes is variable between plant lineages but highly conserved in conifers.

## Results

### Genomic sequences

Genomic sequences were analyzed for several *P. glauca* genes. The sequences were obtained either by targeted BAC isolations, from an early assembly of the *P. glauca* genome [10], or from a sequence capture experiment (for details, see Methods).

A total of 21 BAC clones were isolated each containing a different single copy gene associated with secondary cell-wall formation or with nitrogen metabolism. Following shotgun sequencing by GS-FLX and assembly with the Newbler software, the integrity and identity of each gene was verified. Estimated size of BAC clones was 131 Kb on average and coverage was 144× (for Summary statistics, see Additional file 1: Table S6). Twenty of the 21 targeted genes were complete as determined by sequence alignment indicating full coverage of FL cDNA sequences from spruces and pines (*P. glauca, P. sitchensis, P. taeda* and *Pinus sylvestris*) [25-28] (Additional file 1: Table S7). Nearly all genes were contained within a single contig, except the LIM gene which lacked one exon, and the Susy gene which was complete cDNA sequence but spanned two contigs. None of BACs contained other genes as determined by BLAST searches against the *P. glauca* gene catalog [29] and the Swiss Prot database.

Sequences were also isolated from a whole genome shotgun assembly of *P. glauca*[10]. Sequences with ubiquitous expression were targeted in order to complement the set of more specialized genes which had been selected for BAC isolation. The *P. glauca* genome shotgun assembly was screened with the complete CDS derived from cDNA sequences (according to Rigault et al. [29]) that were highly expressed in most tissues (according to Raherison et al. [30])*.* A total of 18 genomic sequences were randomly selected among those that spanned the entire coding region of the targeted gene*.*

### Gene expression profiles

**Figure 1:**
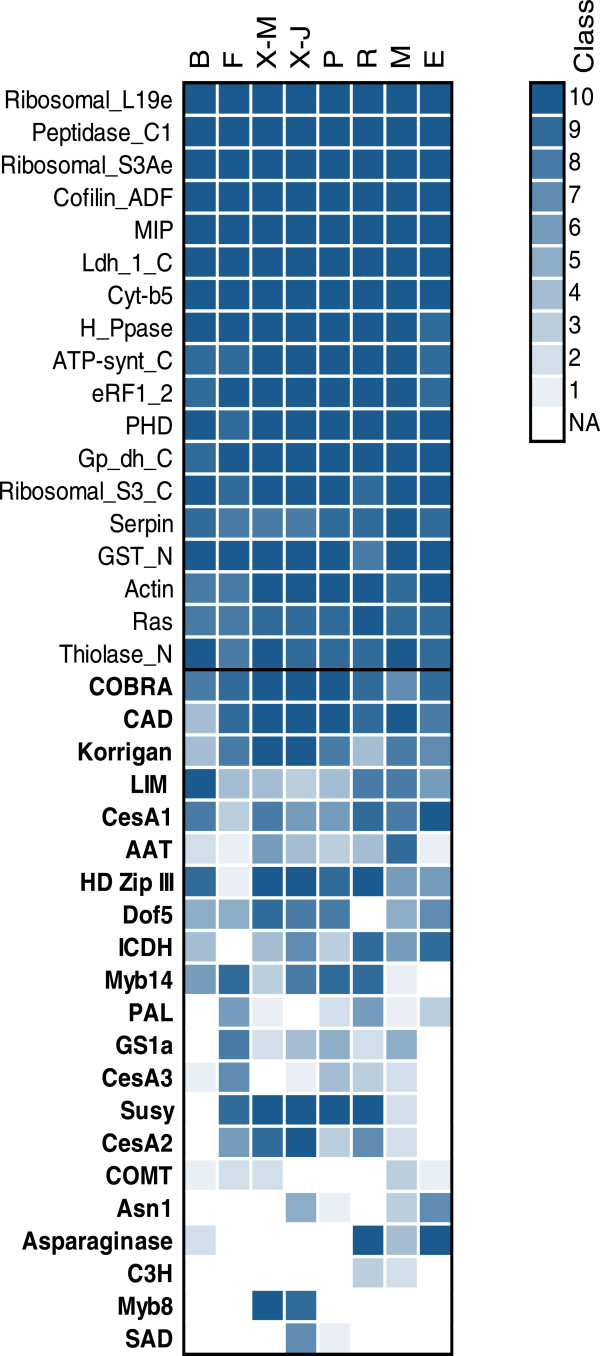
**Transcript accumulation profiles from the PiceaGenExpress database (Raherison et al. [**[30]**]) of the *P. glauca *genes.** The transcript abundance data are classified from 1 to 10, from lowest to highest microarray hybridization intensities detected within a given tissue. The profiles of highly expressed genes (top) (according to Raherison et al. [30]; class 8 to 10) are contrasted with most of the genes associated with secondary cell wall formation and nitrogen metabolism (bottom, names in bold). NA: Not detected. Tissues: B (Vegetative buds), F (Foliage), X-M (Xylem – from mature trees), X-J (Xylem –juvenile trees), P (Phelloderm), R (Adventitious roots), M (Megagametophytes), E (Embryogenic cells).

Transcript accumulation profiles from eight different tissues were obtained from the PiceaGenExpress database [30] for each of the gene sequences described above (Figure 1). The transcript data indicated that the group of highly expressed genes was detected in all tissues and with average abundance class above 9.7 (out of 10) across all tissues (Figure 1, top). In contrast, the genes associated with wood formation and nitrogen metabolism nearly all had tissue preferential expression patterns; they were detected in six tissues on average (range of two to eight tissues) and had an average transcript abundance class of 5.8 in those tissues where the genes were expressed (Figure 1, bottom).

### Gene structures and comparative analysis with angiosperms

The gene structure (exon and introns regions) of *P. glauca* genes was determined by mapping the complete cDNA onto the genomic sequence (BACs or shotgun contigs) for 35 genes. Homologs were retrieved from three well-characterized angiosperm genomes, *Arabidopsis thaliana*[19], *Populus trichocarpa*[18] and *Zea mays*[20]. The comparative analyses considered all of the genes together and also as two separate groups, i.e. genes highly expressed and genes related to secondary cell-wall formation and nitrogen metabolism. On average, the protein coding sequence similarity between *P. glauca* and *A. thaliana* was 76%, 78% with *P. trichocarpa* and 75% with *Z. mays*.

The number of exons and introns was well conserved between homologous genes among the different species (Table 1). The average length of exons was also well conserved between homologs among species (average of 240 bp, median of 155 bp) and varied only slightly between the two sub-groups genes (Table 1 and Additional file 2: Figure S2). Pairwise comparisons of matching exons also indicated conservation of length among the species considered (not shown). These observations indicate that exon structure is generally well conserved.

**Table 1:** Average number and length of exons in genes used for comparative analyses

	**Highly expressed genes**^**1**^			**Secondary cell-wall formation and nitrogen metabolism genes**^**2**^		
	**Exon number**	**Exon length**	**Standard deviation**	**Exon number**	**Exon length**	**Standard deviation**
*Arabidopsis thaliana*	5.9	220.8	215.0	9.1	228.9	189.8
*Populus trichocarpa*	6.2	241.5	253.3	9.4	261.1	263.5
*Zea mays*	6.1	244.5	236.7	9.0	257.6	274.8
*Picea glauca*	6.2	236.5	226.0	9.5	223.9	217.8

^1^Data were obtained from 18 different genes and an average total of 109 exons per species.

^2^Data were obtained from 17 different genes and an average total of 157 exons per species.

**Figure 2:**
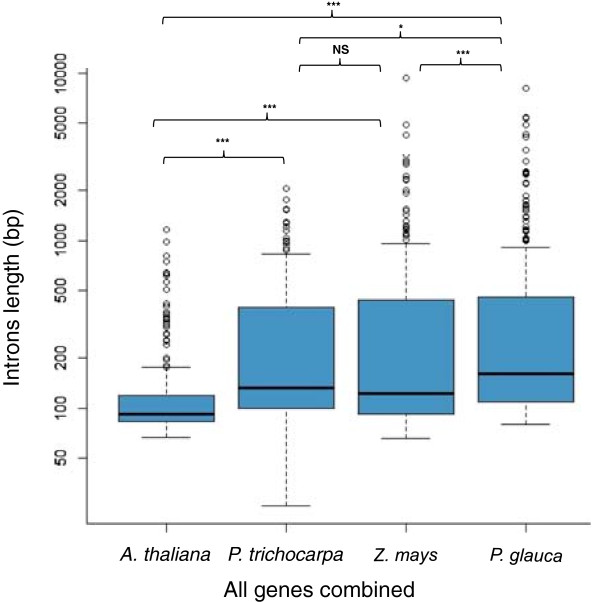
**Comparative analysis of individual intron length in *P. glauca*, *A. thaliana P. trichocarpa *and *Z. mays*.** Box plots represent intron length data for all of the introns of the 35 genes used in comparative analyses. Intron lengths were compared among the four species by Kruskal-Wallis test with post-test analysis by Dunn’s multiple comparisons: NS, not significant (*P* ≥ 0.06); *P = 0.06; **P < 0.01; ***P < 0.001.

In contrast, introns revealed much more variation between species. Our analyses included comparisons of individual introns and of total intronic sequences in each gene. The average length of individual introns (in bp) was 144, 295, 454, and 532 for *A. thaliana, P. trichocarpa*, *Z. mays and P. glauca*, respectively (Figure 2 and Additional file 2: Figure S2). The average intron length varied significantly among *P. glauca* and the three species; pairwise contrasts were significant with *A. thaliana* and *Z. mays,* and nearly significant with *P. trichocarpa* (Figure 2). In *P. glauca*, *P. trichocarpa* and *Z. mays*, we also observed that intron lengths were more heterogeneous as shown by differences between low and upper quartiles, minimum and maximum lengths and outliers of large size (Figure 2). The average length of the longest intron per gene was 382 bp in *A. thaliana*, 806 bp in *P. trichocarpa* ,1652 bp in *Z. mays* and 2022 bp in *P. glauca*.

**Figure 3:**
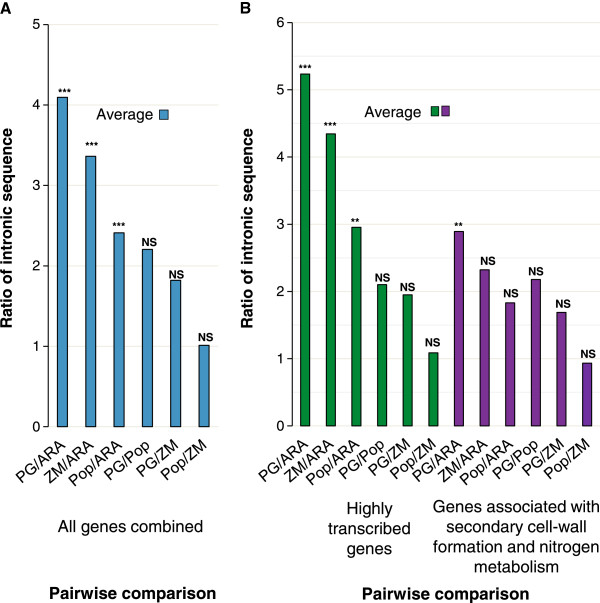
**Comparative analysis of total intron length in *P. glauca*, *A. thaliana, P. trichocarpa *and *Z. mays*.** Average ratio of total length of intron sequences in pair-wise comparisons in: **A**- all genes; **B**- highly expressed genes and genes involved in secondary cell-wall formation and nitrogen metabolism (For individual ratios, see Figure 4). The total intron lengths were compared among the four species by Kruskal-Wallis test with post-test analysis by Dunn’s multiple comparisons: NS, not significant (*P* ≥ 0.05); **P < 0.01; ***P < 0.001.

Comparison of the total length of intronic sequences on a gene-by-gene basis showed that on average, *P. glauca* genes had 4.1 times more intronic sequences than *A. thaliana,* 2.2 times more than *P. trichocarpa* and 1.8 times more than *Z. mays* (Figure 3A). The total length of intron sequences and length ratio was calculated for each gene in pairwise comparisons between all of the species. Comparisons between *P. glauca* and *A. thaliana* gene sets were statistically significant (Figure 3); the ratios were close to five on average in highly expressed genes and three in genes associated with secondary cell-wall formation and nitrogen metabolism (Figure 3B)*.* In contrast, the ratio of total intron lengths between *P. glauca* compared to *P. trichocarpa* and *Z. mays* was constant at around two-fold and the total length of intronic sequence per gene was not statistically different. Results also indicated that *A. thaliana* has significantly less intronic sequence than *P. trichocarpa* and *Z. mays* and that their ratios were most different for the highly expressed genes and more similar for the genes involved in secondary cell-wall formation and nitrogen metabolism (Figure 3B). A significant difference of intron lengths was also observed between the two expression groups within *P. glauca* (p < 0.05).

**Figure 4:**
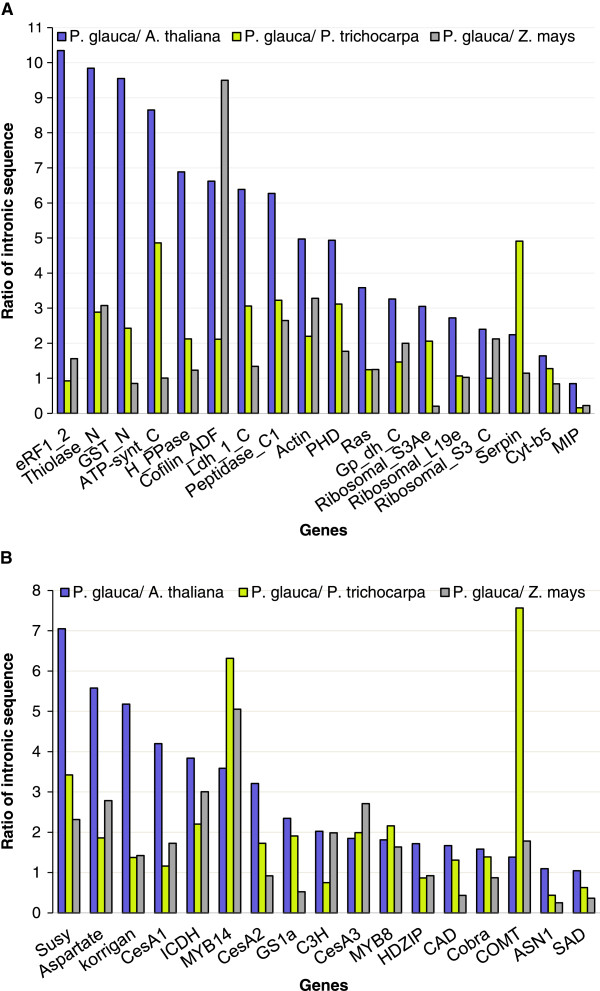
**Gene by gene pair-wise comparisons of total length of intronic sequences in *P. glauca*, *A. thaliana, Populus trichocarpa *and *Z. mays. *(A)** highly expressed genes and **(B)** genes associated with secondary cell-wall formation and nitrogen metabolism.

The variation in the ratios of total intron sequence per genes was quite striking, for both of the gene expression groups (Figure 4). For instance, depending on the gene, the ratios ranged from 0.2 to 10. This high level of heterogeneity in pairwise comparisons is likely to account for the lack of statistically significant differences. In addition, the intron length ratios were not consistent across species (Figure 4A and B).

**Figure 5:**
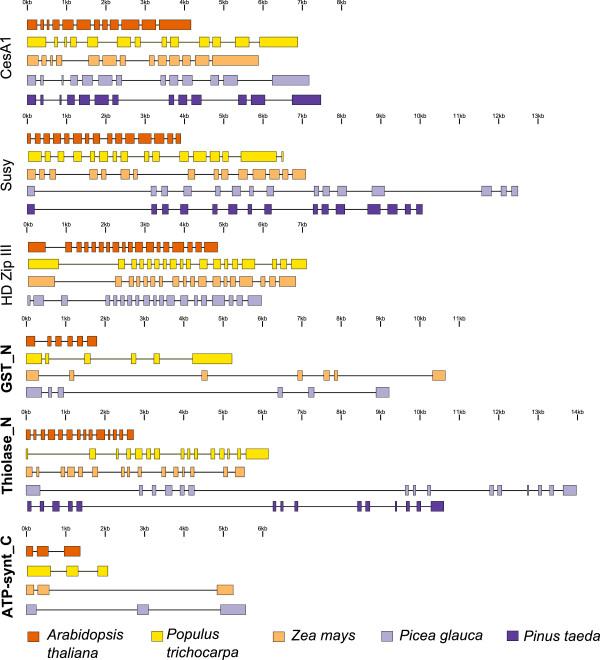
**Gene structure of six genes from different angiosperm and gymnosperm species.** The first three genes are associated with secondary cell-wall formation and nitrogen metabolism; and highly expressed genes are bolded.

In this study, we show that much of the divergence in the total length of intron sequences per gene was related to a few long introns. Very long introns were observed in a few *P. glauca* genes such as PHD, Peptidase_C1 and Thiolase. Structure plots showed that introns in *A. thaliana* generally had uniform lengths whereas the other species had introns that were highly heterogeneous in lengths (Figure 5 and Additional file 3: Figure S3). While most of the *P. glauca* genes only had a few (1–3) very long introns (>1000 bp), gene sequences such as those for sucrose synthase (Susy) had many introns of moderate size (Figure 5). The longest introns in *P. glauca* were most often in a different position than long *Z. mays* and *P. trichocarpa* introns. In addition, we did not observe a trend of increased length in first introns in 5′ UTRs as reported for several eukaryotes [31], as the long introns in *P. glauca* appeared to be randomly distributed.

### Comparative analysis of gene structures between *Picea glauca* and *Pinus taeda*

A total of 23 different genes were submitted to pairwise comparisons between *Picea glauca and Pinus taeda,* which are both of the Pinaceae (for details, see Methods). A high level of similarity was observed for coding sequences (91% on average) indicating that they were likely orthologous genes (Additional file 1: Table S4), and gene structure was conserved between the two conifers, with almost identical numbers of exons. The total intronic sequences per gene did not vary significantly at 3.13 and 3.17 Kbp for *P. glauca* and *P. taeda*, respectively (Additional file 1: Table S1). Pairwise comparison of introns indicated that the majority of individual introns were similar in length in the two species, despite the fact that the two genera diverged ca. 140 million years ago [32,33] (Figure 5). Although these observations are based on a set of only 23 genes, they provide an indication that intron length is mostly conserved between these two conifer genera.

**Figure 6:**
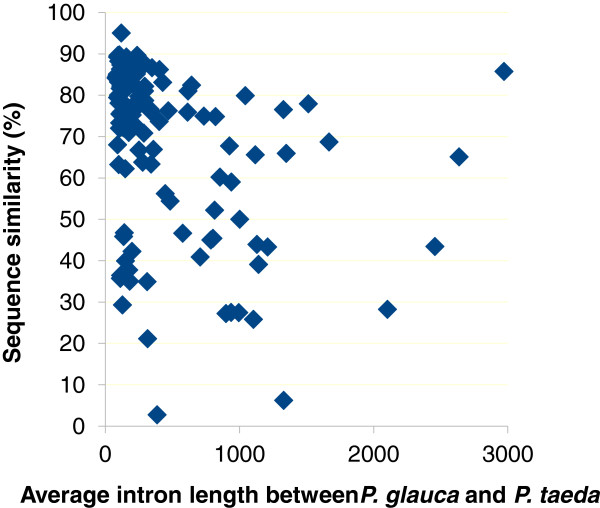
**Relationship between intron size and sequence similarity of introns from *P. glauca *and *P. taeda*.** A total of 138 introns were obtained from 22 genes and sequence alignments were produced with the Needle software (see Methods).

The 138 intron sequences of the 22 genes (PAL gene do not have introns) were aligned between spruce and pine; sequence similarity ranged quite broadly among homologous introns (Figure 6).We observed that highly conserved introns generally were short, and that longer introns had highly variables levels of sequence similarity, except for two introns that were both long and highly conserved.

### Repeat elements in *Picea glauca* genes

The possible origin of long introns as observed in conifer genomes was investigated by searching for the presence of repeated sequences including transposable elements.

First, the repetitive element content of the BACs was estimated based on a repetitive library constructed with *P. glauca* data (see Methods) as a baseline. It was 55% on average, but it varied considerably among the BAC clones, ranging from 18% to 83%. Additional file 4: Figure S1 shows that around half of repetitive sequences were classified as LTR-RT elements and the other half as unknown elements (without significant hits in Repbase and nr genbank).

We then analyzed the sequences of the 35 *P. glauca* genes described above including those identified in BACs, representing a total of 238 introns. The gene structures of these genes were screened for repeat elements using a *P. glauca* repeat library (see Methods). We found repetitive elements in 10 of the genes for a total of 24 unclassified fragments with no significant hits in RepBase; 22 of the fragments produced no hits in genbank and were 179 bp on average and only two had significant hits in nr genbank (Additional file 1: Table S8).

We also extended our analysis to include an additional set of genomic sequences obtained by targeted gene space sequencing based on sequence capture (see Methods, for details). Complete genomics sequences spanning the entire known mRNA sequence were recovered for 5970 complete genes, 1836 of which contained one or more introns. The different repetitive elements identified in introns and exons were then estimated. The proportion of genes harbouring repetitive elements in their introns was 32.4% and was only 3.2% in exons. The repetitive elements represented 2.94% and 0.74% of the intronic and exonic sequences, respectively (Table 2). The repetitive sequences that were identified ranged from 31 to 1142 bp (median 117 bp) in exons and from 17 to 1189 bp (median 114bp) in introns. The unclassified elements were the most numerous, representing on average 80% of the hits in both introns and exons (Table 2). Class I LTR transposons were the most abundant group of classified repetitive elements and were only represented by incomplete elements. The LTRs were accounted for the higher repetitive element sequence representation in introns; however, on average, the sequences identified as Copia and Gypsy elements were longer in exons than in introns.

**Table 2:** **Abundance of repetitive elements in *P. glauca *** **genes obtained from sequence capture**

**Class**	**Exons (%)**	**Introns (%)**
Copia	0.09	0.24
Gypsy	0.09	0.19
LINE	0.03	0.15
UNK^1^	0.03	0.07
NHF^2^	0.49	2.29

A total of 5970 genes were analyzed, 1836 contained one or more introns.

^1^No significant hit in RepBase but significant hits in nr genbank.

^2^No significant hit in RepBase and nr genbank.

## Discussion

This study reports on the detailed gene structure analysis of 35 genes from the conifer *Picea glauca* obtained from BAC sequencing and genome assembly. Recent analyses of the *Picea abies* and *Pinus taeda* genomes have analyzed individual introns and reported among the highest average intron lengths, the longest introns and highest average among long introns [9,12]. We aimed to develop an understanding of the gene structure in conifers through a detailed analysis of entire genes taking into account gene expression profiles, with a particular emphasis on the potential impact of longer introns on gene structure trough comparative analyses. Our findings were also derived from the analysis of nearly 6000 gene sequences obtained from sequence capture sequencing. We present an interpretation of our findings in regard to the evolution of gene structure.

### Evolution of gene structure in plants

Analyses over a broad phylogenetic spectrum in eukaryotes showed that increases in genome size correlate with increases in the average intron length [2,13]. A strong relationship between intron length and genome size was observed from studies in humans and pufferfish [14], species of *Drosophilla*[15], and from studies of plants with small genomes [2,13].

Our study compared the gene structure (introns and exons) of 35 homologous genes between four seed plant species with very different genome sizes. The conifer *P. glauca* has the largest genome with 19.8 Gbp [34]; among angiosperms, the monocot *Z. mays* has a genome of 2.3 Gbp [24], and dicots represent smaller plant genomes in this set, i.e. *P. trichocarpa* with genome of 484 Mbp [18] and *A. thaliana* with the smallest genome of 125 Mbp [19]. In the present study, the average exon length was similar between the four species, but the overall length of genes varied owing to longer introns in *P. glauca, P. trichocarpa* and *Z. mays*. For the set of sequences analyzed, *P. glauca* had 4.1 times more intron sequence per gene than *Arabidopsis*, 2.2 times more than poplar and 1.8 times more than maize (Figures 3 and 4); however, the statistical significance of these differences was variable.

**Figure 7:**
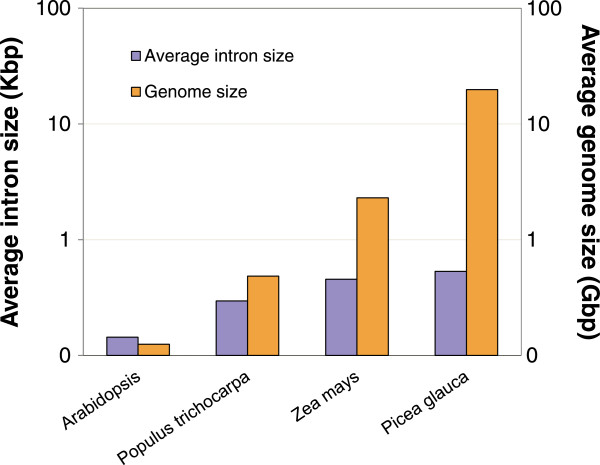
**Variation in introns length and genome size in 35 target genes.** Average intron size for the *Arabidopis*, *P. trichocarpa*, *Z. mays* and *P. glauca* determined from the analysis of 35 homologous genes. Note that Y- axes are in log 10 scale.

The landscape of intron sizes in plants appears rather complex. A significant number of V*itis vinifera* introns were shown to be uncommonly large for its genome size of 416 Mbp, compared to other plants [17]. In *Gossypium,* after multiple inferred rounds of genome expansion and contraction, intron size remained unchanged [16]. Such a pattern may be expected, given that genome size increase by polyploidy is sudden and fundamentally different than other types of genome size variation such as the gradual accumulation or loss of repeat elements over time. Taken together, observations from different plants indicated that events resulting in the expansion or contraction of intergenic regions are not clearly reflected by shifts in introns length. It thus appears that the evolution of intron length and genome size may be uncoupled in plants or alternatively, that the evolution of intron length is lineage specific (Figure 7).

Even though our study was based on 35 genes, our results are consistent with variations of intron size reported for *A. thaliana*, *P. trichocarpa* and *Z. mays* genomes [9,12,18-20]. We concluded that the increased intron length in *P. glauca, P. trichocarpa* and *Z. mays* was heterogeneous compared to *A. thaliana*. Even in genes with many introns, only a few introns were very long, whereas in *Arabidopsis*, genes exhibited a more uniform intron length, suggesting that introns expansion or contraction within a gene may be independent across species.

Comparisons between the *A. thaliana* (125 Mbp) and *A. lyrata* (~200 Mbp) genomes, which diverged about 10 million years ago, showed that most of the difference in genome size was due to hundreds of thousands of small deletions, mostly in noncoding DNA [35]. The authors concluded that evolution toward genome compaction is occurring in *Arabidopsis*. Conifers such as specie*s* of *Picea* and *Pinus* have large amounts of repetitive elements in intergenic regions and apparently more intronic sequence per gene in comparison to many angiosperms. Our results do not reveal whether the *P. glauca* genome and introns are expanding, or alternatively evolving at slower pace, than other plant genomes which are contracting. Some evidence like the presence of very ancient retrotransposon elements [9,36] and the lack of gene rearrangements since before their split from extant angiosperms [8] lend credence to the paradigm that conifer genomes are slowly evolving.

### Repetitive sequences in gene evolution

Transposable elements play a role in plant genes as was shown by the abundance of TE- gene chimeras in *Arabidopsis* which was reported as 7.8% of expressed genes [37]. The abundance of TEs may be especially high in long introns as recently shown in *Picea abies* where most of the introns were longer than 5 Kbp, representing 5% of the total intron count [9]. This trend was also observed in other repeat rich genomes as *V. vinifera* and *Z. mays*[20,21,38].

We isolated *P. glauca* BAC clones each containing a different complete transcription unit for 21 target genes. In each the BACs (average 131 Kb), only one intact gene sequence was identified, which is indicative of large intergenic regions as reported for other conifers [39-41]. Previous studies on conifer trees have considered only two targeted genes (from terpenoid biosynthesis) isolated from *P. glauca* BAC clones [40] and only a few other intact genes with complete coding sequence isolated from BACs in pines [7,39,41].

Complete sequencing of the *P. glauca* BACs showed that the repetitive element content is not distributed uniformly in proximal intergenic regions, as indicated by the variable proportion of repetitive elements among the different BACs. A study in 10 *P. taeda* BACs, sequences similar to eukaryote repeat elements (according to Repbase) represented 23% of the sequence on average, and ranged from 19% to 33% [7]. In *P. glauca*, 26% of BAC sequences were classified as LTR-RT repetitive elements on average and ranged from 8% to 47%, while *P. taeda* had an average of 18.8% of LTR-RT [7]. Furthermore, an average 26% of the *P. glauca* BAC sequences were unknown repeat elements. Results in spruce and pine indicate a relatively low abundance of TEs in gene proximal sequences compared to whole genomes at 70% in the *Picea abies* genome [9] and around 80% in *Pinus taeda*[12].

*Picea* and *Pinus* genomes are reported to have among the highest average for the longest intron per gene, when compared to angiosperms of diverse genome sizes [9]. We verified whether insertions of repetitive elements could be responsible for the length of introns in *P. glauca* in a set of more than 1800 genes sequences, and found that more genes harboring repetitive elements in introns were 10 times more frequent than genes harboring repetitive elements in exons, i.e. 29.8% vs 3.2%. The vast majority of the repetitive elements were short fragments, suggesting that they were remnants or fragments of TE insertions that have not persisted and could represent ancient insertion events. Importantly, interpretation of our findings in *P. glauca* must take into account the fact that the sequences were derived from a sequence capture study and that nearly all of the introns in the data set were <1 Kbp. Thus we show that TE sequences are ubiquitous even in genes that do not harbor long introns, suggesting that their presence has been very widespread during the evolution of conifer genes. An analysis of intact LTR TE in *Picea* genomic sequences showed that most insertions date back to 10 MYA or more, with a maximum around 20–25 MYA [9]. The TE remnants that we detected in *P. glauca* indicate that many genes introns contained TE in a more or less distant past. In this report and in recent analyses of conifer genomes, an emphasis has been place on long introns; however the median intron length in conifers is very similar to other plant species, most of which have a median between 100 bp and 200 bp. Therefore our findings on intron are relevant for a large majority of introns rather than a small fraction represented by large or very large introns.

### Slow evolution of conifer genes

Analyses of the gene structure of 23 orthologous genes between *P. glauca* and *P. taeda* clearly showed the conservation of gene structure and the distribution of intron sizes in spite a divergence time of 100 to 140 MYA [32,33]. The conservation of long introns was also observed across gymnosperm taxa, where a group of long introns in *P. abies* was identified as orthologous to long introns in *P. sylvestris* and *Gnetum gnemon*[9]. We suggest that the long introns observed in *P. glauca* likely date back to a period predating the divergence of major conifer groups. As more conifers genomes become available [9-11] and assembly contiguities are improved it will be possible to extend this analyses of orthologous gene structures among conifers.

We also observed that the sequence of many introns was highly similar between spruce and pine, and that shorter introns were more conserved on average. Between humans and chimpanzee, a strong positive correlation was found between intron length and divergence [42]. The pattern found in conifers as well as observations in primates lead to the hypothesis that shorter introns could be under stronger selection pressure than longer introns, which could be explained by factors such as the maintenance of functional regulatory elements in shorter introns or impacts on RNA transcript processing and stability. In our analysis of sequence similarity between *Picea* and *Pinus*, 20 of the introns were longer than 1 Kbp and only two of them had high sequence similarity. Future studies with more long introns are required to confirm the hypothesis that shorter introns are more conserved in conifers. Despite the fact that introns are assumed to be non-coding, conserved introns may play a functional role related to gene expression.

### Costs and benefits associated with intron size

There is also considerable debate about other factors that may impact the evolution of introns, aside from transposable elements. Lynch [43] stated that the reduced efficiency of selection in regions of low recombination may lead to an increase in intron size if small introns provide a slightly improved transcription efficiency or splicing accuracy. On the other hand, Comeron and Kreitman [3] proposed that there might be situations in which a longer intron is selectively advantageous as an explanation for intron persistence and increased lengths. If so, there would be indirect selection for large introns in regions of low recombination because they can reduce the load caused by deleterious mutations by increasing the recombination rate. It was proposed that conifers have low recombination rates at both the genome and within-gene scales [44]. Their low recombination rates may explain at least in part, the accumulation of longer introns.

The high degree of sequence conservation that we observed in short introns between spruce and pine may also depend on the recombination rate within genes, where small introns would be under stronger selection because of efficiency in transcription and splicing, and long introns in regions of low recombination diverge because of reduced selection pressure. Another factor underlying the evolution of intron size is that intron length would be constrained by energy use during transcription, given that large introns represent a higher cost of transcription, the so-called “economy” or low-cost transcription hypothesis [4]. In the present study, the 35 *P. glauca* genes analyzed were divided in two groups based on their expression profiles, i.e. 17 genes associated with secondary cell-wall formation or nitrogen metabolism, many of which had tissue preferential expression, and 18 genes that were highly transcribed in a large range of tissues (based on Raherison et al. [30]). The highly expressed genes had more intronic sequences per gene on average than the more specialized subset of genes (4,182 bp versus 3,013 bp). We also observed a large variation among genes in each group, i.e. from 446 to 12,009 bp in highly transcribed genes and 440 to 9,847 bp in the set of more specialized genes. These observations do not support the “economy” hypothesis in *P. glauca* as there appears to be no clear rule governing the relationship between intron size and expression levels or profiles. In humans, genes contained total intronic sequences are ~5,500 bp per gene on average [45], which more than any plant described so far. It was observed that intron length declines steadily as the expression level increases in humans, in agreement with the low-cost transcription hypothesis [4]. Considering the smaller amount of intron sequences in plant genes including conifers compared to humans, it may be that the economy rule does not impact their introns as strongly as in vertebrates and that other evolutionary forces are main drivers of intron size evolution. This interpretation is consistent with the findings reported for the *P. abies* genome [9]. Future studies with more genes are needed to confirm this hypothesis.

## Conclusions

Our results indicate that *P. glauca* has longer introns than *Arabidopsis*, *P. trichocarpa* and *Z. mays* on average due the presence of few long introns. Intron size and the position of long introns within genes were variable between plant lineages but well conserved in conifers. Our findings are consistent with recent reports indicating that conifers accumulate very long introns but we point out that long introns represent a relatively small fraction of the overall intronic content, which is reflected by the median length of similar size to other plants. We show that RE sequences are detected at a high frequency (32%) even in introns <1 Kbp, indicating their ubiquitous presence in conifer genes over the course of evolution.

Taken together, our observations and the recent literature suggest that the evolution of plant gene structure is determined by more interacting forces than classically expected. The pattern is reminiscent of the heterogeneity of rates of evolution at the genetic, genomic and morphological levels seen among seed plants including angiosperms, conifers, annual and perennial taxa. It stands to reason that the distinctive features of the conifer genome, such as its large size and relatively small occupancy of the gene space, its conserved macro-structure, the large numbers of repetitive elements, and long introns, represent the product of the intricate evolutionary history of conifers.

## Methods

### *Picea glauca* BAC isolation and validation

A BAC library developed from the single *Picea glauca* (Moench) Voss individual PG29 from BC Ministry of Forests was utilized. The non-arrayed library consisted of approximately 1.1 million BAC clones with an average insert size of 140 kbp, representing approximately 3× coverage of *P. glauca* genome [40]. The library was screened by quantitative PCR (qPCR) through successive steps using BAC super-pools and pools, serial dilutions and clone identify verification by amplicon sequencing. The BAC isolation and sequencing are reported here for the first time.

We isolated 21 BAC clones each containing a different single copy gene from *P. glauca* (see list of genes and accession numbers in Additional file 1: Table S2). Each of the genes screened was represented by a unique FL-cDNA clone in *P. glauca* as described in Rigault et al. [29]. The selected genes encoded enzymes and transcriptional regulators involved in secondary cell-wall formation and nitrogen metabolism and were subject to manual curation. They were chosen as to facilitate comparison with BAC isolation studies conducted in other conifers species (e.g. [21]). Two sets of gene specific primers were designed for each gene based on the cDNA sequence available in *P. glauca* gene catalogue [29]. The genomic sequence obtained was used to design two additional primers such that two small amplicons of 120–200 bp could be amplified by quantitative PCR (qPCR) (Additional file 1: Table S3). All of the primer sets were verified by PCR and qPCR using the genomic DNA from *P. glauca,* genotype PG-653, and then they were used to screen the BAC library in three steps. See PCR conditions in Additional file 5.

The BAC library was subdivided into pools with a titer 1000 BACs on average, which were arrayed into ten 96 deep-well plates. Each plate was inoculated in 96-well culture plates with 1 ml of terrific broth (TB) and 20 μg/mL of chloramphenicol and grown in a 37°C shaker at 300 rpm overnight. The same TB medium and growing conditions were utilized to culture bacteria throughout the screening steps. Bacterial cultures from each of the columns and rows within a plate were combined in a total of 200 super-pools for DNA isolation as described in Osoegawa et al. [46].

The first step followed Jeukens et al. [47]. Briefly, the super-pool DNA was amplified by the two small amplicons for each target gene by qPCR using QuantiTect SYBR Green master mix as described in Boyle et al. [48]. The intersection of a positive row and a positive column was indicative of positive wells on the original plate. The presence of target genes in the positive super-pools was verified by qPCR in 30 μL reactions using QuantiTect SYBR Green master [48]. We performed PCR of the long amplicon and its purification for gene sequence validation by Sanger sequencing (Additional file 1: Table S3). The second step of the screening relied on serial dilutions of the super-pools to inoculate 50 bacteria from the positive per well in a 96 deep-well plate. DNA super-pools were extracted and screened by qPCR using the same conditions as in the first step. Then, we extracted DNA from 1 μL of bacterial culture from each well of a positive column to test it by qPCR and determine the positive well in the column. From the positive well of the same bacterial culture plate, we proceeded with serial dilutions and we inoculated a 96 deep-well plate with one isolated colony per well. The third step of the screening consisted to pool columns and rows of bacterial cultures. We identified positive wells by qPCR and plated the culture of each positive well on a different Petri dish and one colony per dish was inoculated in 5 mL TB with chloramphenicol. DNA was extracted from 2 mL of each culture. Positive isolated clones were validated by PCR, qPCR and resequencing of the long amplicon. The validation steps to confirm gene identity and integrity proved essential as conifers such as conifers contain many pseudogenes that reduce the efficiency of targeted BAC isolation [39].

The 21 isolated BAC clones, each identified by screening for a different gene (for accessions numbers, see Additional file 1: Table S2) were sequenced by Roche 454 FLX pyrosequencing at McGill University and Genome Québec Innovation Centre, Montreal, Canada. Sequences were assembled *de novo* into contigs using the GS *De novo* Assembler module of Newbler version 2.3 (Roche) [49]. In this analysis, the BAC vector and *E. coli* genome were trimmed and the assembly parameters were a minimum overlap of 200 bp length, minimum overlap identity of 98% and minimum contig length of 500 bp. In general, more than one contig per BAC was obtained; therefore, the order of the contigs within each BAC was tested by PCR.

To determine gene structure (introns and exons), cDNAs were mapped onto the BAC contigs containing the respective gene using est2genome incorporated in the annotation software MAKER [50]. Four of the genes were eliminated from the comparative gene structure analyses because they were either incomplete, lacked introns or identifiable homologs in the species targeted for comparative analyses.

### *Pinus taeda* orthologous sequences

Seven BAC clones of *Pinus taeda* containing orthologs of *P. glauca* genes were identified by BLAST [51] using an e-value threshold of 1e-20 and sequence identity > 90% (Additional file 1: Table S4). An additional 16 sequences were identified by BLAST [51] using an e-value threshold of 1e-20 in the whole genome shotgun assembly of *Pinus taeda*[11]. Their gene structures were defined using est2genome [50] and *P. taeda* cDNA or *P. glauca* cDNA when *P. taeda* complete cDNA was not available. Accessions numbers available in Additional file 1: Table S4. A pairwise alignment of all corresponding intron and exon sequences of orthologous genes between *P. glauca* and *P. taeda* was conducted, followed by the estimation of their similarity with the software Needle, part of the analysis package EMBOSS [52]. The BAC clones containing the LIM gene in *P. glauca* and the Korrigan, Peptidase_C, Thiolase, Gp_dh_C and eRF1_2 genes in *P. taeda* lacked an intron and exon; these missing exons and introns were excluded from the comparison between *P. glauca* and *P. taeda*.

### Screening for highly expressed genes in whole genome shotgun assembly

Based on transcript profiles (PiceaGenExpress database [30]) a set of 500 gene sequences each representing a unique FL-cDNA clone that was highly expressed in all tissues was identified from the *P. glauca* gene catalog [29]. A preliminary assembly of the *P. glauca* genome assembly described by Birol et al. [10] was screened with each of these sequences. The screening was performed by exonerate and est2genome model [53], which considers intron/exon boundaries. The cDNA/genome alignments were further filtered based on the identity and length coverage to retain only complete alignments with entire cDNAs; i.e., genes with complete genomic sequence. We randomly selected 18 the genomic sequences thus identified as containing complete structures of highly expressed genes. As for genes contained into the BACs, genes annotations were generate automatically and curated manually individual reciprocal BLASTs and sequence alignments.

### Identification of closest homologs in angiosperms

Homologous sequences to *P. glauca* genes were identified in *Arabidopsis thaliana and P. trichocarpa* by BLASTX [51] with threshold e-value of 1e-10. Reciprocal analysis (BLASTX) between the *A. thaliana, P. trichocarpa* sequence and the *P. glauca* gene catalogue was used to verify that the genes were the closest homologs among known sequences. In *Zea mays*, the closest homolog was identified based on *A. thaliana* sequences by BLASTX, with a threshold e-value of 1e-10, and orthology was verified in the Maize Genome Project Sequencing database (Additional file 1: Table S5). We also performed a BLASTX of *P. glauca* against *Z. mays* sequences and we verified that the closest homologs identified between *Z.mays* and *A. thaliana* were among the best hits. Gene structures were recovered from the databases of TAIR 10 [54], Phytozome (JGI v3.0 gene annotation of assembly v3 of *P. trichocarpa*) [18,55] and the Maize Genome Sequencing Project [56]. Accession numbers available in Additional file 1: Table S5.

From the 21 genes contained into the *P. glauca* BAC clones, four genes were eliminated from the gene structure comparative analyzes between *P. glauca*, *A. thaliana, P. trichocarpa* and *Z. mays*: (1) Dof5 because a clear A*. thaliana* homolog could not be identified, (2) asparaginase because a clear *Z. mays* homolog could not be identified, (3) PAL because it lacked introns and (4) LIM because it presented an incomplete sequence (cDNA). A total of 35 genes had their gene structure compared with closest homologs in angiosperms: 17 genes related to secondary cell wall formation and nitrogen metabolism and 18 highly transcribed genes with little tissue-specific expression.

### Statistical analyses of introns

Intron lengths were compared between *P. glauca*, *A. thaliana*, *P. trichocarpa* and *Z. mays* by nonparametric Kruskal-Wallis tests with post-test analysis by Dunn’s multiple comparisons, because intron length did not follow a Gaussian distribution. Comparisons of two groups of genes used by Wilcoxon rank sum test with continuity correction; this test was used to compare total intron sequences in *P. glauca* genes belonging to the two expression groups and to compare *P. glauca* with *P. taeda*. Data analyses were performed using the R packages coin and multcomp [57-59].

### Gene space obtained from sequence capture technology

Sequences were obtained by using genomic DNA hybridizations on a custom *P. glauca* chip containing oligonucleotide baits for 23,864 genes. The method development, the DNA sequence isolation and analysis procedure and the resulting sequence data are reported in this manuscript for the first time.

DNA was extracted from needles of the *P. glauca* individual 77111 from the Canadian Forest Service as described in Pelgas et al. [60] using the DNeasy Plant mini kit according to the manufacturer’s instructions (QIAGEN). One microgram of high quality DNA was used to prepare a GS-FLX rapid library according to the manufacturer instructions (Roche). The library was amplified by ligation-mediated PCR using 454 A and B primers as described in the NimbleGen SeqCap EZ Library LR User’s guide.

Custom probes were designed by Nimblegen based on the cDNA sequences and ESTs from the *P. glauca* gene catalogue [29]. We used a Newbler (gsAssembler module v2.5.3) assembly of sequences from random genomic sequencing (0.15× of coverage) from *P. glauca*[29] to identify highly repetitive elements and to filter out probes representing such elements as they were expected to reduce the efficiency of the sequence capture. Next, a comparative genomic hybridization (Array CGH) experiment was conducted in collaboration with Nimblegen (Madison, WI, USA) to eliminate probes with abnormally high level of hybridization that could not be identified with *in silico* approaches. Throughout the process, probes within genes harboring abnormally high capture levels were eliminated. The final design covered 23,864 genes. The target enrichment procedures including quantitative PCR assessments are described in Additional file 5.

Emulsion PCR and GS-FLX Titanium sequencing was performed according to manufacturer’s instructions at the Plateforme d’Analyses Génomiques of the Institut de Biologie Intégrative et des Systèmes (Université Laval, Quebec, Canada). Raw sequencing reads were *de novo* assembled using the gsAssembler module of Newbler v2.5.3. Contigs were screened for complete gene structures based on the *P. glauca* gene catalogue [29]. Technical details are available in Additional file 5.

### *Picea glauca* repetitive library and identification of repeat elements

For repeat identification, a random sample of 100,000 *P. glauca* 454 reads from randomly sheared DNA was searched *de novo* for repeats using the software RepeatScout [61]. The results were filtered by removing low complexity sequences and sequences shorter than 100 nt, and retaining only repeats having at least 10 matches when mapped onto the original 454 set using RepeatMasker [62]. Since RepeatScout is tailored to analyze complete genomes or at least large scaffolds, its output is usually fragmented when the program is run on random sheared reads. In order to reduce fragmentation, we merged the repeats belonging to the same element running cap3 [63] under relaxed settings (-o 30, -p 80, -s 500) on the RepeatScout output. Finally, the entire set of repeated sequences was clustered using the software cd-hit-est [64] by collapsing all the repeats sharing at least 80% similarity in order to remove redundancies.

Repeat characterization proceeded by similarity searches were used to associate candidate repeats to known TE families and to remove repeats showing similarity to gene sequences and being possibly part of gene families. In particular, the repeat candidates from each species were searched against RepBase [65] using TBLASTX [57] and setting as significance threshold an e-value of 1e-5. Repeats that did not provide significant hits were used as queries in BLASTX searches against the non-redundant (nr) division of Genbank. Those having significant hits with genes were removed from the library while those having significant hits with TEs were labeled accordingly and the remaining repeats were considered as unclassified.

The search for repeat elements in all BAC contigs and in the gene space obtained from sequence capture was conducted using RepeatMasker [62] using the *Picea glauca* repetitive library and default parameters.

## Competing interests

The authors declare that they have no competing interests.

## Authors’ contributions

KR and CR provided the *P. glauca* BAC library; IG and BB ran the BAC isolation experiments; BB performed sequence capture experiments and assembled the sequences; PR analyzed the sequence capture sequences and mapped them to the cDNA models; IB, SJ, JBoh, JBou and JM participated in the assembly of *P. glauca* genome; JS conducted the data analysis and interpretation of data and results, and drafted the manuscript; AZ developed the *P. glauca* repetitive library; JBou and JM contributed to the supervision and discussion of the research; JM, JBou and KR revised the manuscript. All of the authors approved the manuscript.

## Supplementary Material

Additional file 1: Table S1 Gene structure data of orthologs of *Picea glauca* and *Pinus taeda*. **Table S2.** List of genes associated with secondary cell-wall formation or with nitrogen metabolism in *P. glauca* targeted for BAC isolations. **Table S3.** Primer information and sequences used for BAC screening and sequencing validation. **Table S4.** Accession numbers of *P. taeda* orthologs and sequence similarity to *P. glauca*. **Table S5.** Accession numbers for the closest homologous sequences between *P. glauca*, *Arabidopsis thaliana*, *Populus trichocarpa* and *Zea mays*. **Table S6.** Summary of sequencing results of *P. glauca* BAC clones isolated each containing a different single copy gene associated with secondary cell-wall formation or with nitrogen metabolism*.***Table S7.** GenBank accessions of complete cDNA utilized for gene structure definition when the cDNA in *Picea glauca* gene catalogue was incomplete. **Table S8.** Repetitive elements detected within gene structure of the 35 *P. glauca* genes. Click here for file

Additional file 2: Figure S2 Comparative analysis of individual intron length in *P. glauca*, *A. thaliana, P. trichocarpa* and *Z. mays*. A. Average and median length of individual introns in all genes. B Average and median length of individual introns in highly expressed genes and genes associated with secondary cell-wall formation and nitrogen metabolism in four species. Intron lengths were compared among the four species by Kruskal-Wallis test with post-test analysis by Dunn’s multiple comparisons: NS, not significant (P > 0.06); * P < 0.06; **P < 0.01; ***P < 0.001. Click here for file

Additional file 3: Figure S3 Boxplot of the 35 homologous genes in *P. glauca*, *A. thaliana*, *P.trichocarpa* and *Z. mays.* Click here for file

Additional file 4: Figure S1 Content of repetitive elements in 21 different BAC clones. The analysis used the RepeatMasker software and a *P. glauca* repetitive sequence library (see Methods). Repetitive elements were classified as LTR (long terminal repeat) and unclassified (no hit in RepBase). Click here for file

Additional file 5 **Supplemental file.** Additional experimental procedures for BAC isolation and sequence capture. Click here for file
