# Supplementary material for: Evolution of gene structure in the conifer Picea glauca: a comparative analysis of the impact of intron size
Source: BMC Plant Biol. 2014 Apr 16;14:95. doi: 10.1186/1471-2229-14-95 (PMC4108047; doi:10.1186/1471-2229-14-95)
Supplement: Additional file 1: Table S1 — Gene structure data of orthologs of Picea glauca and Pinus taeda. Table S2. List of genes associated with secondary cell-wall formation or with nitrogen metabolism in P. glauca targeted for BAC isolations. Table S3. Primer information and sequences used for BAC screening and sequencing validation. Table S4. Accession numbers of P. taeda orthologs and sequence similarity to P. glauca. Table S5. Accession numbers for the closest homologous sequences between P. glauca, Arabidopsis thaliana, Populus trichocarpa and Zea mays. Table S6. Summary of sequencing results of P. glauca BAC clones isolated each containing a different single copy gene associated with secondary cell-wall formation or with nitrogen metabolism.Table S7. GenBank accessions of complete cDNA utilized for gene structure definition when the cDNA in Picea glauca gene catalogue was incomplete. Table S8. Repetitive elements detected within gene structure of the 35 P. glauca genes. [file 1471-2229-14-95-S1.pdf]

Supplemental table 1. Gene structure data of orthologs of *Picea glauca* and *Pinus taeda*.

	Picea glauca		*Pinus taeda*		
Gene	N° exons	Total introns length (bp)	N° exons	Total introns length (bp)	Ratio of intron length PG/PT
LIM1	5	1877	5	2018	0,9
CesA1	13	3843	13	4340	0,9
CesA2	14	5462	14	7242	0,8
PAL	1		1		
Korrigan	5	1256	5	1183	1,1
Susy	15	9847	15	7424	1,3
MYB8	4	464	4	525	0,9
CAD	6	1159	6	2395	0,5
COBRA	6	1733	6	1777	1,0
COMT	3	1384	3	578	2,4
C3H	3	1446	3	1600	0,9
GS1a	14	3648	14	3680	1,0
H_PPase	8	5220	9	5239	1,0
Ldh_1_C	7	5433	7	3163	1,7
Cyt-b5	2	1613	2	1723	0,9
Gp_dh_C	11	3269	11	3951	0,8
Serpin	2	1380	2	1276	1,1
Peptidase_C1	9	1784	9	1798	1,0
eRF1_2	2	350	2	349	1,0
Ribosomal_S3_C	6	2618	7	3791	0,7
Cofilin_ADF	3	2687	3	2855	0,9
Ras	8	2964	8	3942	0,8
Thiolase_N	14	9491	14	9059	1,0
Average	7,0	3133,1	7,1	3177,6	1,0

Supplemental table 2. Genes associated with secondary cell-wall formation or with nitrogen metabolism in *P. glauca* targeted for BAC isolations.

**Genes**	**GenBank accession**	***Picea glauca* Reference ID (GCAT-pgl[1])**	***Picea glauca* BAC GenBank accessions**
Aspartate Aminotransferase (AAT)	BT117995*	GQ03919_P11*	KC860233
Asparagine synthetase (Asn1)	CO478951*	GQ0177_K02*	KC860234
Asparaginase	BT101939	GQ0133_M14	KC860235
Coumarate 3-hydroxylase (C3H)	BT106474	GQ03002_I06	KC860236
Cinnamyl alcohol dehydrogenase (CAD)	BT112280	GQ03312_O11	KC860237
Cellulose synthase (CesA1)	BT116636	GQ03803_L08	KC860238
Cellulose synthase (CesA2)	BT106827	GQ03011_H12	KC860239
Cellulose synthase (CesA3)	BT116976	GQ03810_K09	KC860240
COBRA	BT104865	GQ02816_A06	KC860241
Caffeate o-methyltransferase (COMT)	BT108042	GQ03116_D16	KC860242
Dof5	BT105779	GQ02828_F13	KC860243
Glutamine synthetase (GS1a)	BT114315	GQ03512_F02	KC860244
Homeobox-leucine zipper family protein (HD-ZIPIII)	BT117426*	GQ03819_E16*	KC860245
Isocitrate dehydrogenase (ICDH)	BT104232	GQ02808_B04	KC860246
Korrigan	EX433116*	GQ03912_H23*	KC860247
LIM 1	BT117230	GQ03815_F15	KC860253
Myb14	BT101254*	GQ0082_F08*	KC860248
Myb8	BT108136*	GQ03117_E18*	KC860249
Phenylalanine ammonia-lyase (PAL)	BT100475*	GQ0015_I17*	KC860250
Cinnamyl alcohol dehydrogenase (SAD)	BT112656	GQ03319_B08	KC860251
Sucrose synthase (Susy)	EX336506*	GQ03002_P04*	KC860252

* Incomplete cDNA in the white spruce gene catalogue (GCAT-pgl[1]) and GenBank. See Supplemental table 6

[1] Rigault et al. 2011

Supplemental table 3. Primer information and sequences used for BAC screening and sequencing validation

					Amplicon length (bp)[3]		
Gene name	Primer 1 [1]	Primer 2 [2]	Primer 3 [1]	Primer 4 [2]	1-4	1-2	3-4
COBRA	TACCAATACCAACATGAGGGTCCAG	TGTTATGTTGCCGTTTGGATCTAGTG	ACCAGGGACTCCTTACAACCAACAG	TGGTTCCTGCATTACCCACACTTAC	500	136	125
CAD	TTGCCATCTGCAAGCAATACAGTAG	TCCTGCATTTTTAGATGTACCTGAGAG	TGCATCATACCAGCAATGGGTATAG	ACAACGCCCAGCATAACTAGCTTTC	1750	176	197
Korrigan	ATGCTCAGGTTGGGAAAGGAGATAC	CAGGTCGGAACAAGAACTGCAATC	CCTGCCTTCCGTTCCTTCAATAG	CTTCCGTGGTTCAACTGAATCAAAC	894	128	152
CesA1	ATCGTCTATCCCCTCACGTCTCTTC	CACATTATGAATCAATCTGAGTTGGAG	GTTGGTGATCAACCTTGTTGGAATG	GCAGGACAGACCACAATATAACGATG	1463	127	194
AAT	ATTGATTCTTTGTTCCCCTTCCAAC	AATTTCAGCTTGCTGGATAGATCAG	CCAAAGCACTTTGTTACAGCTTGTG	TACTGCTAGCACCTGATGTGGTCTG	1022	225	285
HD Zip III	ACACAAGCTGTGAGTCTGTGGTGAC	CCGATAATTTGGAAAGGTAGACCAATG	ACACCTGTTTCCATTGATCCCTTTC	TGAGTACCACTCAGGGACCTTTCAC	996	122	130
Dof5	AGTGAAGCTGGGCTCCTTGAAATAC	CACTGGTGTAACCAAGTCAAGGCTAAG	TTCTTTTGTGTGTGCAAACAACCTG	GACAGTAATGGGGTTTTGCCTCTTC	603	139	143
ICDH	TGATGGTGATGTGCAGAGTGATTTC	TCTTACCAGTACAGACGTCATCAAG	TCTTGGATTTTGTTGTCTTGTCCAG	ATGCTGTTCGTGCTAGTTTCACCTC	1214	220	129
Myb14	CGGACAACGAGATAAAGAACCACTG	GAGGGCCAGGTAATGTTACTGTTATG	CAGAATGCGGGGTTTCTCTCATTAC	CTGGACCGCCAGCGATAGTAAG	589	124	168
PAL	AGCTGCCTTTAAGAGGAACCATCAC	CTCCGCTCATTTCTGTTCCATCTC	AGCGATCATGGAGTATGTCTTGGAC	GCAGATCTGATTACCTCGACCTGAG	529	126	156
GS1a	TTGCAAATCGAGGAGCTTCAGTTAG	ATATGTCCAATTTTGCAATCAACATC	ATAGGGCGATGGCTTTAAAAGACTG	CAGCTAAAAAGCGCAACAACATTTC	1853	139	182
CesA3	ATCCTCAGGTTGGGAGAAAAGTCTG	CAGTTCCCACATATACTGGCCCTTG	TGTGGATATGAAGACAAAACCGAATG	TCGTTTGGGCATACAGTAAATGGAC	1475	155	174
Susy	ATATTGATCCCTACCATGGGGTTTC	GGAATCAGGCCCTGTACTTACTTTTC	GGGTATTATAGTCATCGTGGCCTGTTC	CTTCAACCCCATTTACGCTTTCTTC	692	110	135
CesA2	CAGGGTCCAGTGTATGTAGGGACTG	GCTTTTTGGATGATTTCTTTGTTTTC	GGTTAATGTCGCAGAAGAGCTTTG	CTCGTATCCGCAGCTTATGACG	169	151	1227
COMT	GAGGGGTTTAAGTCTGGGGAGAAG	GCAATCACATGAGGAAGATCGAAG	GCGGAACCCAAGTCACAATATTG	GTAACCTCCAAAACGCTGAAATGTG	984	131	177
Asn1	GGGCTAATAAGTCGACATCTGCATG	TAAGGATTCTGCTCCTCGTCAAATG	TCTAGGTGTCCATGCCTCCGCATAC	CGGGTGCCTATACTTTCTTCCATTC	1013	265	120
Asparaginase	GGGTTGTGTTGTTGTTGACAGTGAG	GCATAAGTGCCTGCCCCTATTATTG	TACAAACAAGGGTTTGTCCTTGGTC	AATTGAACCCATAACGGGCAGATAC	648	120	139
C3H	TTTGTGGATGCATTGCTCACTCTAC	ATGGGTATGAGGAACCCCTTAATTC	GGCCTGAGAGATTTATTGAGGAAGATG	CATTCGAAATGATGAAGGAGGTGTC	572	152	154
Myb8	AGCTCCGAGTCGATCTGTAGGTTTC	GTACATGGATGGATTCGCCATTATC	GAAATGCTGCCTCCGTTTTCAAG	GGAGTACTGAGCATTGTGGTCCTG	696	148	218
SAD	TTAAAGACTCCTCCGCTCTTCGTTC	ACCGGCTTAATTCTCCTTCATTCAC	CTCCCACCTTTGGAGGTTACTCATC	GGAGGTATTTCATGGGGCTGTAGAC	933	141	233
LIM	CGAGGGCTCAAGACCGTTATTTTAC	TTGCAATGGTTACATCTGAAGCAAG	TGCCCTGAACTGGTTAATAAAGTTG	CTGCGTTTTCAAAGATAATGGCAAG	572	152	154

[1] 5’ primer
[2] 3’ primer
[3] Amplicons obtained from pairs 1-2 and 3-4 were used for PCR screening and verification of BAC; amplicons obtained from primer pair 1-4 were used for Sanger sequencing to verify the identity of the gene.

Supplemental table 4. Accession numbers of *P. taeda* orthologs and sequence similarity to *P. glauca*.

A. BAC clones of *P. taeda.* Sequence identity with *P. glauca* was based on coding sequence.

Genes	***P. taeda* BAC GenBank accession**	**cDNA accession**	**Sequence identity with *P. glauca* (%)**
Cellulose synthase (CesA1)	AC241295.1	AY789650.1	93
Cellulose synthase (CesA2)	AC241331.1	AY789651.1	92
LIM 1	AC241349.1	BT117230*	93
Myb8	AC241314.1	DQ399057.1	93
Korrigan	AC241332.1	EF619968.1	90
Phenylalanine ammonia-lyase (PAL)	AC241300.1	PTU39792	90
Sucrose synthase (Susy)	AC241289.1	EF619967.1	93

* *P. glauca* cDNA was utilized because *P. taeda* cDNA was incomplete

B. Sequence from *P. taeda* shotgun assembly (Wegrzyn et al., 2014). Identity with *P. glauca* was based on coding sequence.

**Genes**	**cDNA accession**	**Sequence identity with *P. glauca* (%)**
Serpin	BT100637*	92
C3H	AY064170.1	93
COBRA	BT104865*	92
Peptidase_C1	BT107363*	91
eRF1_2	BT107692*	91
COMT	BT108042*	87
Gp_dh_C	BT111068*	89
Cofilin_ADF	BT111103*	93
Ras	BT111640*	93
Ribosomal_S3_C	BT112106*	86
CAD	Z37991.1	92
Gs1a	BT103460*	88
H_Ppase	BT115473*	90
Thiolase_N	BT115978*	89
Ldh_1_C	BT117871*	92
Cyt-b5	BT119045*	85

* *P. glauca* cDNA was utilized because *P. taeda* cDNA was incomplete

Supplemental table 5. Accession numbers for the closest homologous sequences between *P. glauca*, *Arabidopsis thaliana*, *Populus trichocarpa* and *Zea mays*.

Picea glauca		*Arabidopsis thaliana*		*Populus trichocarpa*		*Zea mays*	
GCAT accessions	GenBank accessions	GenBank accessions	Identity %	JGI v3.0 gene name	Identity %	GenBank accessions	Identity %
GQ0033_L20	BT100637	NP_175202.1	49	Potri.014G036000.1	55	NP_001167655.1	52
GQ0082_F08	BT101254	NP_195574.1	65	Potri.009G134000.1	60	NP_001106009.1	66
GQ0177_K02	CO478951	NP_196586.1	82	Potri.005G075700.1	81	ACF80883.1	76
GQ0182_H10	BT102359	NP_564098.2	95	Potri.009G125000.1	90	NP_001150274.1	94
GQ02808_B04	BT100366	NP_176768.1	85	Potri.004G074900.1	85	NP_001140324.1	77
GQ02810_I18	BT104452	NP_568336.1	70	Potri.017G125100.1	61	NP_001151414.1	60
GQ02816_A06	BT104865	NP_568930.1	83	Potri.015G060000.1	85	NP_001105970.1	71
GQ03002_I06	BT100373	NP_850337.1	76	Potri.006G033300.1	82	NP_001130442.1	69
GQ03002_P04	EX336506	NP_199730.1	78	Potri.002G202300.1	76	NP_001105194.1	78
GQ03011_H12	BT106827	NP_199216.2	80	Potri.004G059600.1	76	NP_001105672.1	85
GQ03012_N11	BT106885	NP_187818.1	96	Potri.006G192700.1	97	NP_001150462.1	97
GQ03104_C22	BT107197	NP_171777.1	91	Potri.015G029500.1	93	NP_001148813.1	93
GQ03106_H10	BT107363	NP_563648.1	61	Potri.002G184200.1	64	NP_001150152.1	68
GQ03109_L23	BT107587	NP_195193.1	89	Potri.005G051200.1	88	NP_001149096.1	86
GQ03111_J03.2	BT107692	NP_189295.3	93	Potri.014G141000.1	95	NP_001151538.1	93
GQ03116_D16	BT108042	NP_200227.1	42	Potri.019G102900.1	46	NP_001149617.1	36
GQ03117_E18	BT108136	NP_172425.2	65	Potri.010G004300.1	79	NP_001132070.1	83
GQ03210_A11	BT109562	NP_197551.2	74	Potri.018G050200.1	83	NP_001169011.1	64
GQ03232_E11	BT111068	NP_187062.1	89	Potri.001G335800.1	86	NP_001105385.1	90
GQ03232_K24	BT111103	NP_567182.1	76	Potri.001G106200.1	72	NP_001151716.1	73
GQ03301_J24	BT111640	NP_171715.1	77	Potri.004G226600.1	80	NP_001105441.1	77
GQ03310_B15	BT112106	NP_198403.1	85	Potri.006G222100.1	87	NP_001149150.1	91
GQ03312_O11	BT112280	NP_195149.1	68	Potri.009G095800.1	70	NP_001105654.1	69
GQ03319_B08	BT102039	NP_195643.1	67	Potri.009G062800.1	61	NP_001147726.1	60
GQ03512_F02	BT103460	NP_568335.1	81	Potri.017G131100.1	83	ACB06727.1	80
GQ03610_A06	BT115139	NP_567178.1	85	Potri.003G128600.1	84	NP_001104934.1	84
GQ03617_H21	BT115473	NP_173021.1	84	Potri.006G063000.1	85	NP_001105380.1	82
GQ03709_L23	BT115978	NP_199583.1	80	Potri.014G168700.1	83	NP_001148667.1	75
GQ03803_L08	BT106211	NP_199216.2	64	Potri.011G069600.1	73	NP_001105672.1	63
GQ03810_K09	BT116956	NP_197244.1	74	Potri.006G181900.1	75	NP_001105532.1	76
GQ03819_E16	BT117426	NP_174337.1	62	Potri.001G372300.1	73	NP_001142394.1	65
GQ03912_H23	EX433116	NP_199783.1	74	Potri.003G151700.1	79	NP_001183308.1	77
GQ03915_D23	BT117871	NP_171936.1	89	Potri.010G071000.1	89	NP_001147160.1	86
GQ03919_P11	BT100655	NP_850022.1	67	Potri.005G079200.	72	NP_001143769.1	74
GQ04013_M05	BT119045	NP_190458.1	63	Potri.012G137800.1	66	NP_001149328.1	65

Supplemental table 6. Summary of sequencing results of *P. glauca* BAC clones isolated each containing a different single copy gene associated with cell- wall formation or with nitrogen metabolism.

BAC	Total of contigs	Average coverage	Total size (bp)
Asparagine synthetase (Asn1)	2	135	130054
Asparaginase	5	497	39145
Aspartate Aminotransferase (AAT)	9	141	192670
Coumarate 3-hydroxylase (C3H)	14	70	161072
Cinnamyl alcohol dehydrogenase (CAD)	5	140	104305
Cellulose synthase (CesA1)	14	91	133796
Cellulose synthase (CesA2)	8	89	150078
Cellulose synthase (CesA3)	6	117	114058
Cobra	4	188	75244
Caffeate o-methyltransferase (COMT)	7	167	104865
Dof5	13	78	196708
Glutamine synthetase (GS1a)	6	67	141000
Homeobox-leucine zipper family protein (HD-ZIPIII)	14	287	101367
Isocitrate dehydrogenase (ICDH)	5	56	118357
Korrigan	5	119	83070
LIM1	11	89	137136
MYB14	14	204	160947
MYB8	4	73	92372
Phenylalanine ammonia-lyase (PAL)	4	111	148199
Cinnamyl alcohol dehydrogenase (SAD)	6	51	112140
Sucrose synthase (Susy)	12	227	135947
Average	8	143	125359

Supplemental table 7. GenBank accessions of complete cDNA utilized for gene structure definition when the cDNA in *Picea glauca* gene catalogue was incomplete.

Genes	***Picea glauca*** **Reference ID (GCAT-pgl[1])**	**Specie**	**GenBank accession**	**Reference**
Aspartate Aminotransferase (AAT)	GQ03919_P11	*P. sitchensis*	WS0284_A12	Ralph et al. 2008
Asparagine synthetase (Asn1)	GQ0177_K02	*P. sylvestris*	AJ496567	Canas et al. 2006
Homeobox-leucine zipper family protein (HD-ZIPIII)	GQ03819_E16	*P. glauca*	HQ391914	Cote et al. 2010
Korrigan	GQ03912_H23	*P. sitchensis*	WS02912_I08	Ralph et al. 2008
Myb14	GQ0082_F08	*P. glauca*	pending	Fortin et al. (unpublished)
Myb8	GQ03117_E18	*P. taeda*	DQ399057	Bedon et al. 2007
Phenylalanine ammonia-lyase (PAL)	GQ0015_I17	*P. glauca*	pending	This report
Sucrose synthase (Susy)	GQ03002_P04	*P. taeda*	EF619967	Nairn et al. 2008

[1] Rigault et al. 2011

Supplemental table 8. Repetitive elements detected within gene structure of the 35 *P. glauca* genes[1].

Gene	Number of Matches	Matching Class[2]	Average lenght (bp)
AAT	3	NHF	105
CAD	2	NHF	86
CesA1	3	NHF	152
CesA2	7	NHF	162
CesA2	1	UNK	103
Korrigan	1	NHF	243
H_Ppase	1	NHF	190
Thiolase_N	1	NHF	331
GST_N	2	NHF	325
Peptidase_C1	2	NHF	217
Ldh_1_C	1	UNK	121

[1]All of the Repetitive elements were detected in intron sequence.

[2]Repetitive elements are classified as NHF (no significant hit in RepBase and nr genbank) and UNK (significant hits in nr genbank only).
