# Supplementary material for: Evolution of gene structure in the conifer Picea glauca: a comparative analysis of the impact of intron size
Source: BMC Plant Biol. 2014 Apr 16;14:95. doi: 10.1186/1471-2229-14-95 (PMC4108047; doi:10.1186/1471-2229-14-95)

Supplemental figure 2. Comparative analysis of individual intron length in *P. glauca*, *A. thaliana, P. trichocarpa* and *Z. mays*. A. Average and median length of individual introns in all genes. B Average and median length of individual introns in highly expressed genes and genes associated with secondary cell-wall formation and nitrogen metabolism in three species. Intron lengths were compared among the three species by Kruskal-Wallis test with post-test analysis by Dunn's multiple comparisons: NS, not significant (P>0.06); * P<0.06; **P<0.01; ***P<0.001.

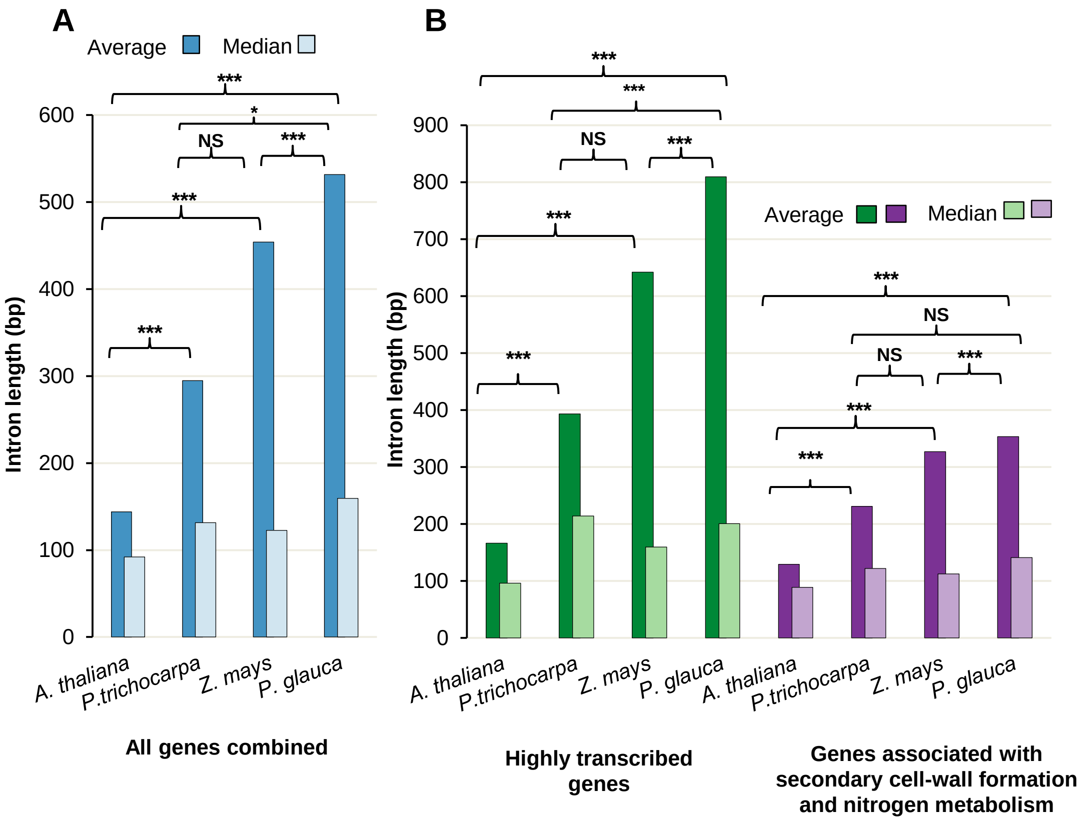

Supplement: Additional file 2: Figure S2 — Comparative analysis of individual intron length in P. glauca, A. thaliana, P. trichocarpa and Z. mays. A. Average and median length of individual introns in all genes. B Average and median length of individual introns in highly expressed genes and genes associated with secondary cell-wall formation and nitrogen metabolism in four species. Intron lengths were compared among the four species by Kruskal-Wallis test with post-test analysis by Dunn’s multiple comparisons: NS, not significant (P > 0.06); * P < 0.06; **P < 0.01; ***P < 0.001. [file 1471-2229-14-95-S2.pdf]
