# Supplementary material for: Evolution of gene structure in the conifer Picea glauca: a comparative analysis of the impact of intron size
Source: BMC Plant Biol. 2014 Apr 16;14:95. doi: 10.1186/1471-2229-14-95 (PMC4108047; doi:10.1186/1471-2229-14-95)
Supplement: Additional file 3: Figure S3 — Boxplot of the 35 homologous genes in P. glauca, A. thaliana, P.trichocarpa and Z. mays. [file 1471-2229-14-95-S3.pdf]

Supplemental figure 3. Boxplot of the 35 homologous genes in *P. glauca*, *A. thaliana*, *P.trichocarpa* and *Z. mays*

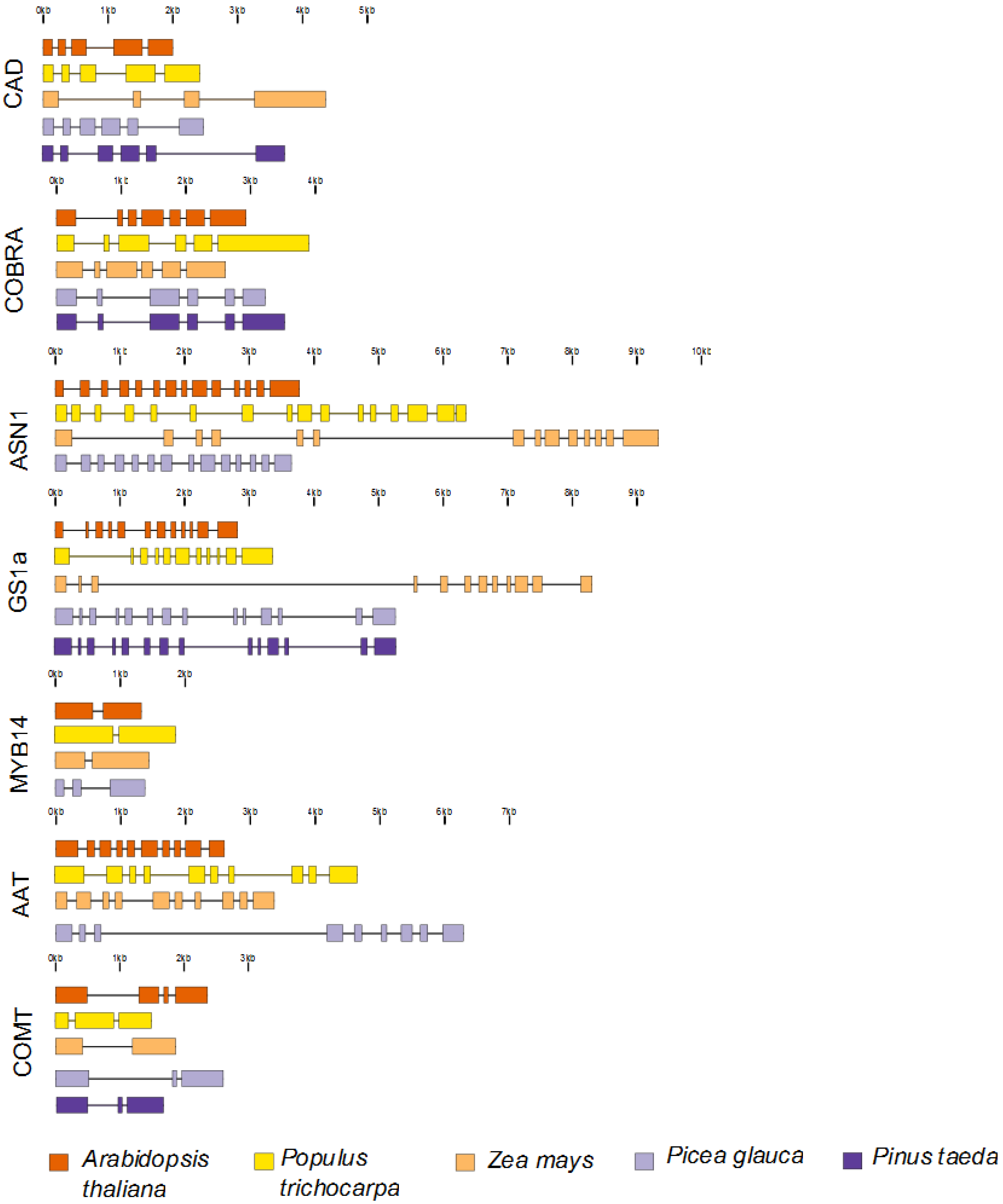

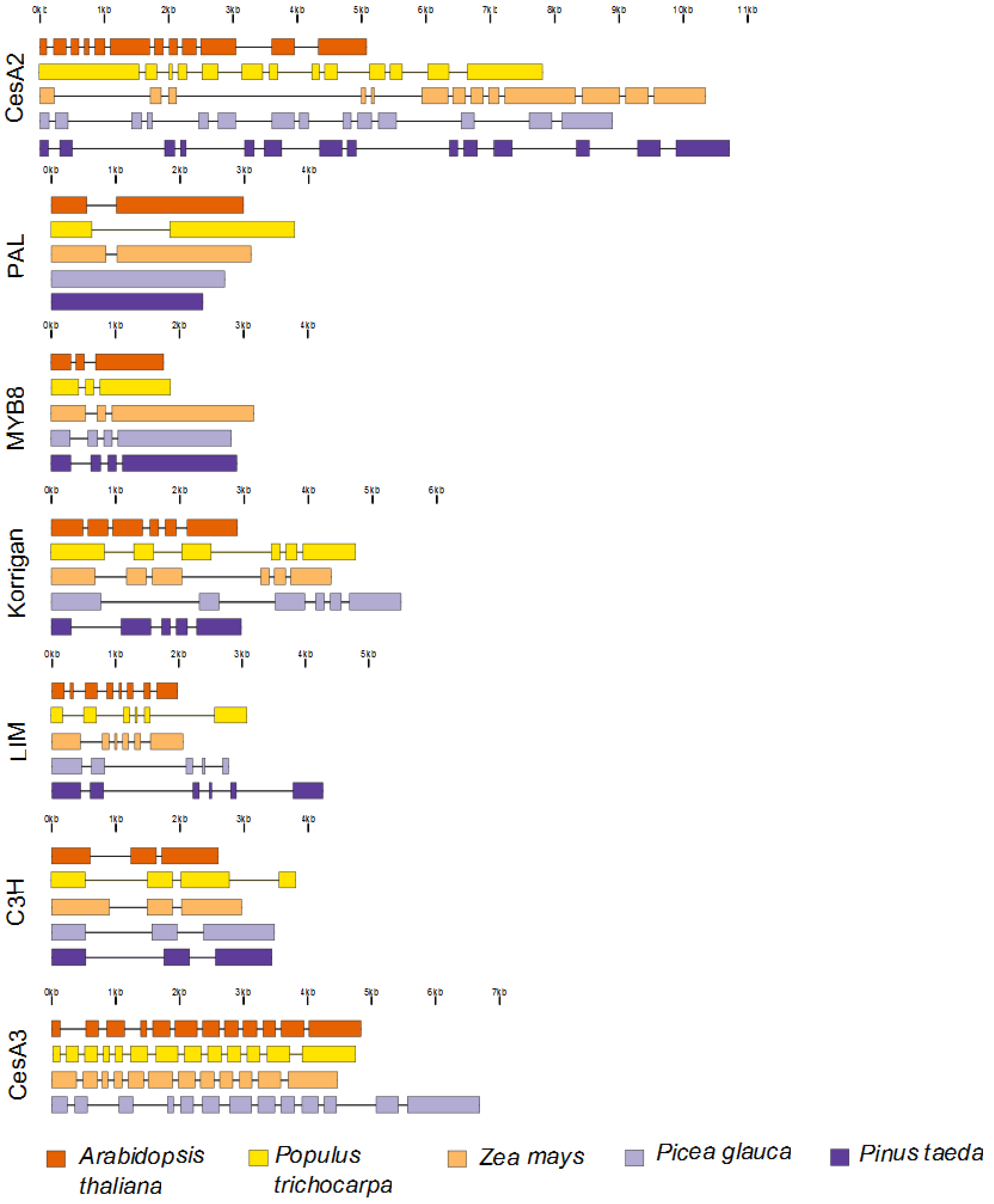

CesA2
PAL
MYB8
Korrigan
LIM
C3H
CesA3
0kb
1kb
2kb
3kb
4kb
5kb
6kb
7kb
8kb
9kb
10kb
11kb
Arabidopsis thaliana
Populus trichocarpa
Zea mays
Picea glauca
Pinus taeda

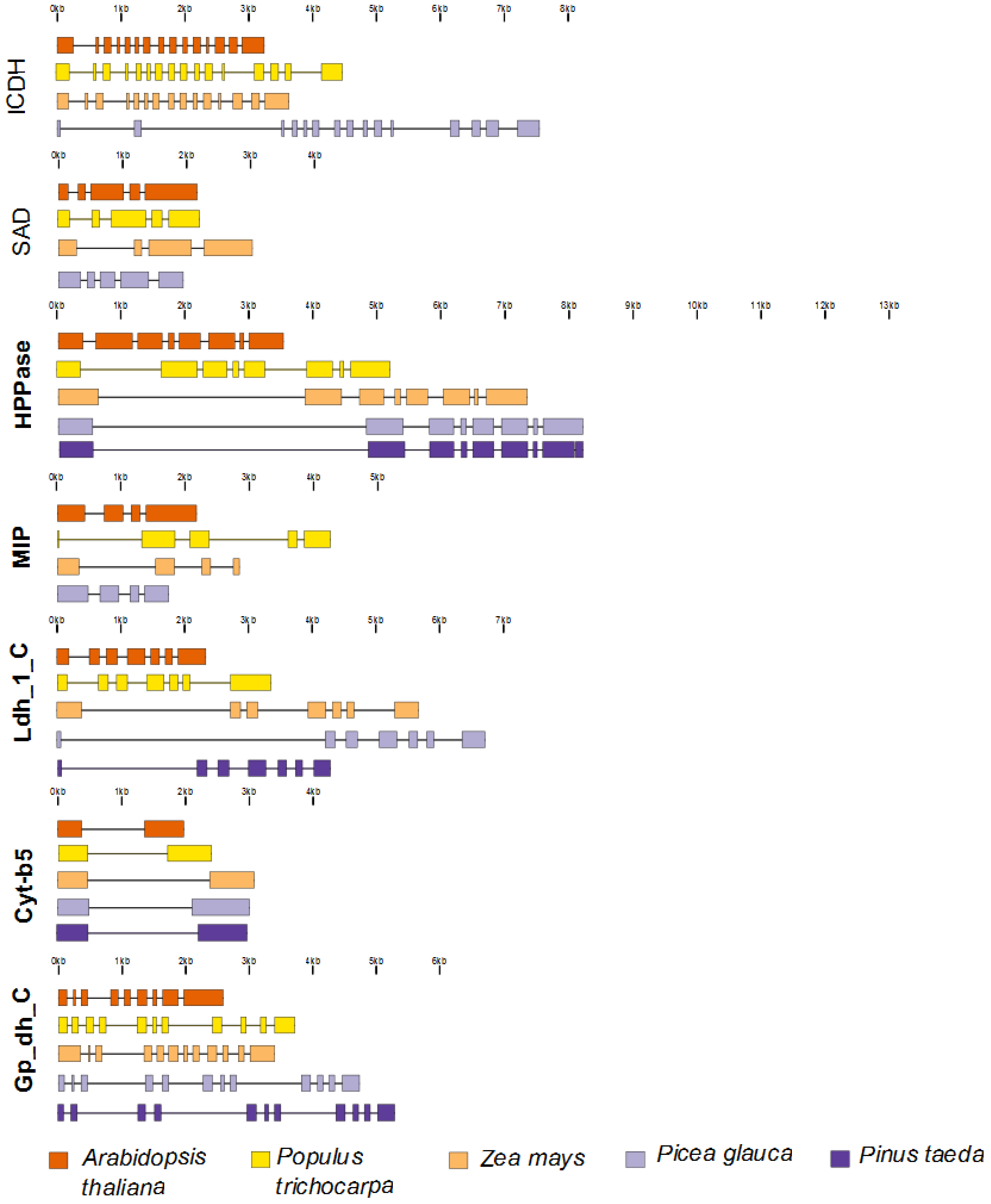
ICDH
SAD
HPPase
MIP
Ldh_1_C
Cyt-b5
Gp_dh_C
Arabidopsis thaliana
Populus trichocarpa
Zea mays
Picea glauca
Pinus taeda

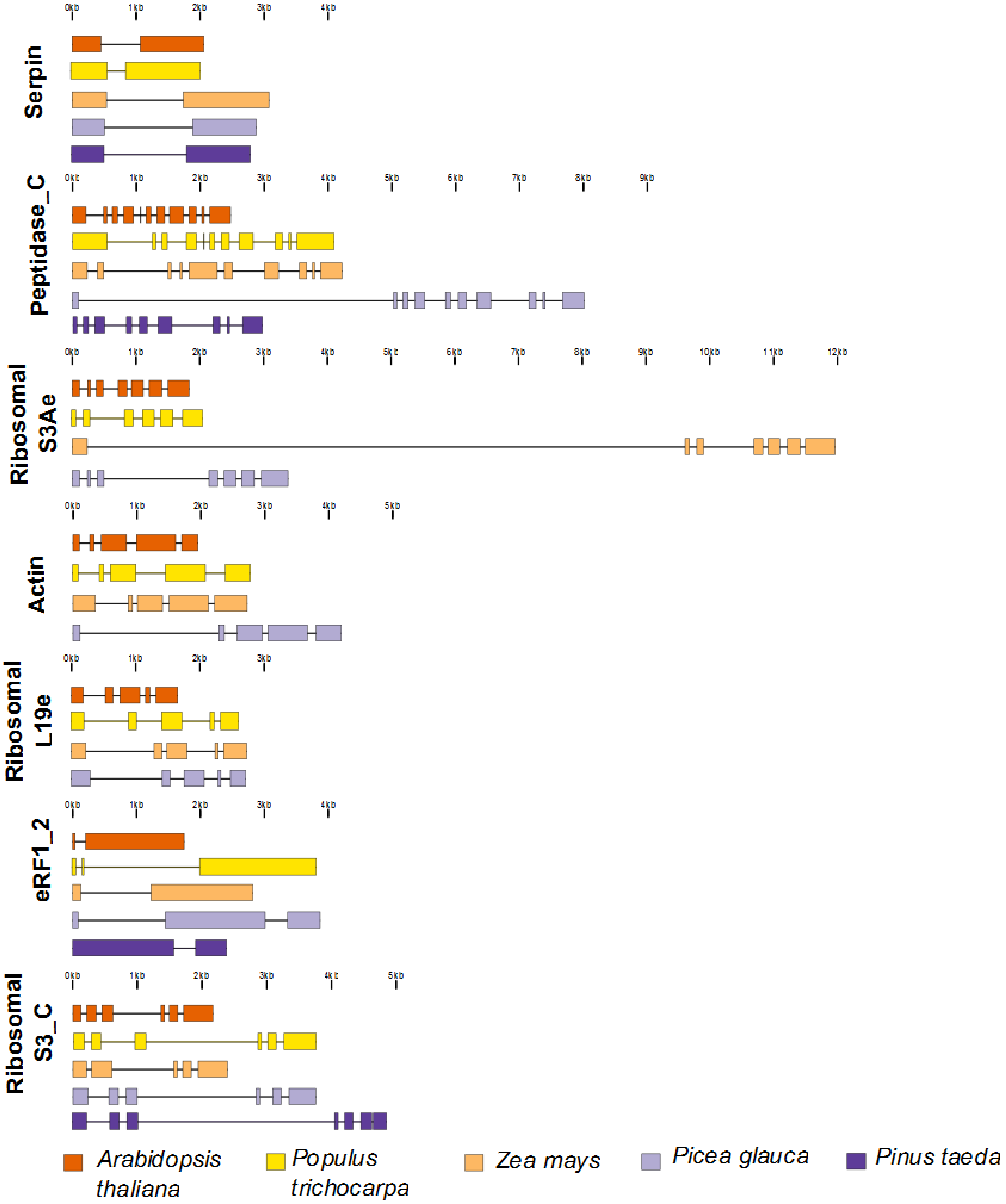

Serpin
Peptidase_C
Ribosomal S3Ae
Actin
Ribosomal L19e
eRF1_2
Ribosomal S3_C
0kb
1kb
2kb
3kb
4kb
5kb
6kb
7kb
8kb
9kb
10kb
11kb
12kb
Arabidopsis thaliana
Populus trichocarpa
Zea mays
Picea glauca
Pinus taeda

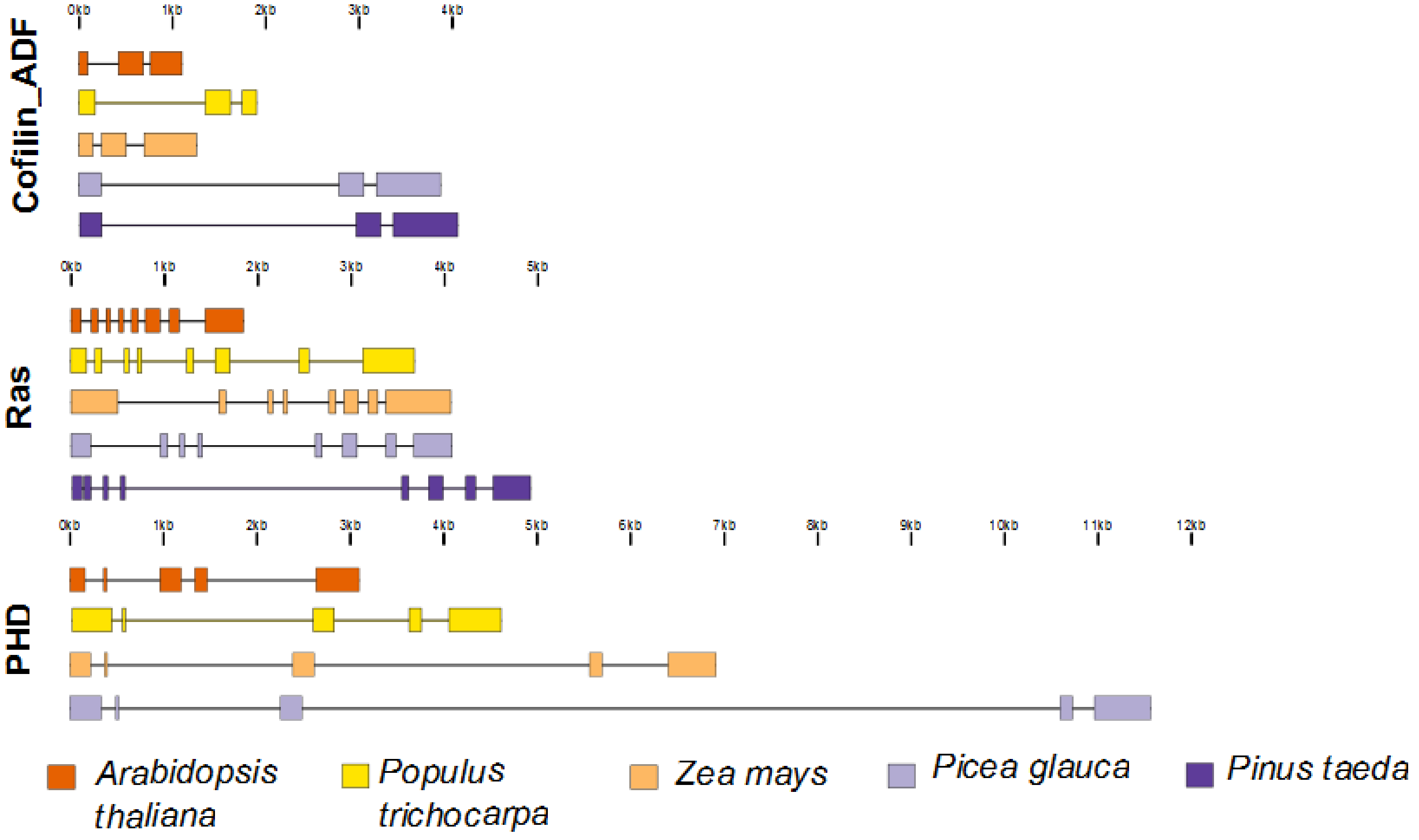
Cofilin_ADF
Ras
PHD
0kb
1kb
2kb
3kb
4kb
5kb
6kb
7kb
8kb
9kb
10kb
11kb
12kb
Arabidopsis thaliana
Populus trichocarpa
Zea mays
Picea glauca
Pinus taeda
