# Supplementary material for: Evolution of gene structure in the conifer Picea glauca: a comparative analysis of the impact of intron size
Source: BMC Plant Biol. 2014 Apr 16;14:95. doi: 10.1186/1471-2229-14-95 (PMC4108047; doi:10.1186/1471-2229-14-95)

Supplemental figure 1. Content of repetitive elements in 21 different BAC clones. The analysis used the RepeatMasker software and a *P. glauca* repetitive sequence library (see Methods). Repetitive elements were classified as LTR (long terminal repeat) and unclassified (no hit in RepBase).

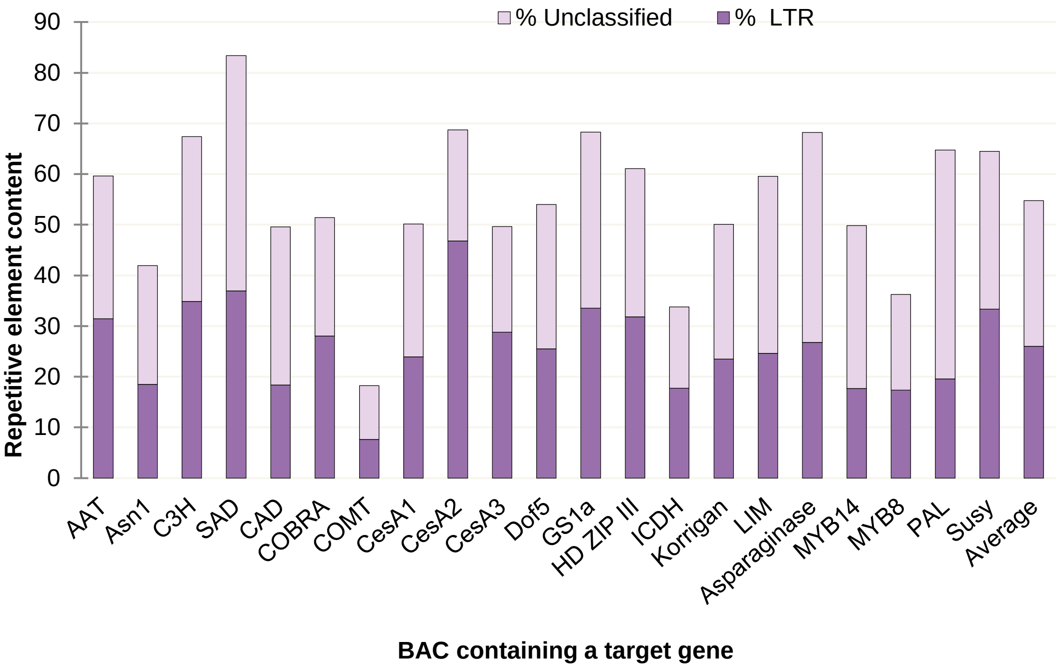

Supplement: Additional file 4: Figure S1 — Content of repetitive elements in 21 different BAC clones. The analysis used the RepeatMasker software and a P. glauca repetitive sequence library (see Methods). Repetitive elements were classified as LTR (long terminal repeat) and unclassified (no hit in RepBase). [file 1471-2229-14-95-S4.pdf]
