# Supplementary material for: Evolution of gene structure in the conifer Picea glauca: a comparative analysis of the impact of intron size
Source: BMC Plant Biol. 2014 Apr 16;14:95. doi: 10.1186/1471-2229-14-95 (PMC4108047; doi:10.1186/1471-2229-14-95)
Supplement: Additional file 5 — Supplemental file. Additional experimental procedures for BAC isolation and sequence capture. [file 1471-2229-14-95-S5.pdf]

Supplemental file. Additional experimental procedures for BAC isolation and sequence capture.

PCR conditions for *Picea glauca* BAC isolation and validation

The first set of primers was designed so as to obtain one long amplicon of 500-1000 bp by PCR. PCRs of the long amplicon were carried out in 50 µL reactions using Platinum ® Taq DNA polymerase High Fidelity (Life Technologies, Carlsbad, CA, USA) 0.2µM of each primer, 2 µl of genomic DNA] and carried out following the following conditions: 5 min activation at 95 °C followed by 35 cycles consisting of 15 s at 95 °C, 1 min at 62 °C and 1 min at 68°C; to finish 5 min at 68°C. The PCR products were purified using QIAquick PCR purification kit (Qiagen, Germantown, MD, USA) following manufacturer’s instructions. The primers sequences are available in supplemental table 2.

Gene space obtained from sequence capture technology

Genomic DNA hybridization and target enrichment

Target enrichment was performed by using 2.1M developer arrays (Roche Nimblegen, Madison, WI, USA) with the SeqCap hybridization and wash kit (Roche Nimblegen, Madison, WI, USA) following the guidelines in the Sequence Capture Array delivery user’s guide with the exception that we used a plant capture enhancer as previously described [65,66]. Briefly, a plant capture enhancer (Roche Nimblegen, Madison, WI, USA) and hyb enhancing A and B primers were added to one microgram of amplified *P.*

glauca library and dried. The mixture was resuspended in 1X SC hybridization buffer containing SC component A and heated to 70 °C for 10 minutes to rehydrate. The mixture was incubated at 95 °C for 10 minutes and brought to 42 °C prior to be loaded on the capture array. The hybridization was carried out at 42 °C and its duration was extended to 72 hours given the very large size of the *P. glauca* genome. Non-captured DNA was washed away according to the manufacturer's instructions. The captured DNA was amplified by ligation-mediated PCR using 454 A and B primers as described in the NimbleGen SeqCap EZ Library LR User's guide.

Target enrichment using SeqCap EZ developer (Roche Nimblegen, Madison, WI, USA) was performed according to the general guidelines provided in the NimbleGen SeqCap EZ Library LR User's guide. Briefly, 10 µl of plant capture enhancer (Roche Nimblegen, Madison, WI, USA) and 5 ul of 100 µM of hyb enhancing A and B primers were added to one microgram of amplified library and dried. The mixture was resuspended in 7.5 µl of 2X SC hybridization buffer and 3 µl of SC component A and heated to 70 °C for 10 minutes. After a quick spin, 4.5 µl of capture oligonucleotides solution in water were added and the hybridization mixture was incubated at 95 °C for 10 minutes followed by 72 hours at 47.5 °C. The hybridization mixture was put in contact with Streptavidin coated Dynabeads (Invitrogen, Carlsbad, CA, USA) and non-captured material was washed away according to the NimbleGen SeqCap EZ Library LR User's guide. The captured DNA was amplified by ligation-mediated PCR using 454 A and B primers as described in the NimbleGen SeqCap EZ Library LR User's guide. The quality of the captures was assessed by comparing pre- and post-capture libraries with quantitative PCR (qPCR) and primers designed against four spruce ESTs. The primer-pair

efficiency brought to the power of the Cq difference between post- and pre-capture generated fold enrichment. These values varied from gene to gene but were around 100 times, on average.

References

1. Haun WJ, Hyten DL, Xu WW, Gerhardt DJ, Albert TJ, Richmond T, Jeddeloh JA, Jia G, Springer NM, Vance CP, Stupar RM: **The composition and origins of genomic variation among individuals of the soybean reference cultivar Williams 82**. *Plant Physiol* 2011, **155**:645–655.

2. Bolon Y-T, Haun WJ, Xu WW, Grant D, Stacey MG, Nelson RT, Gerhardt DJ, Jeddeloh JA, Stacey G, Muehlbauer GJ, Orf JH, Naeve SL, Stupar RM, Vance CP: **Phenotypic and genomic analyses of a fast neutron mutant population resource in soybean**. *Plant Physiol* 2011, **156**:240–253.
